# Method to assess the temporal persistence of potential biometric features: Application to oculomotor, gait, face and brain structure databases

**DOI:** 10.1371/journal.pone.0178501

**Published:** 2017-06-02

**Authors:** Lee Friedman, Mark S. Nixon, Oleg V. Komogortsev

**Affiliations:** 1 Department of Computer Science, Texas State University, San Marcos, Texas, United States of America; 2 Department of Electronics and Computer Science, University of Southampton, Southampton, United Kingdom; Universidad Autonoma de Madrid, SPAIN

## Abstract

We introduce the intraclass correlation coefficient (ICC) to the biometric community as an index of the temporal persistence, or stability, of a single biometric feature. It requires, as input, a feature on an interval or ratio scale, and which is reasonably normally distributed, and it can only be calculated if each subject is tested on 2 or more occasions. For a biometric system, with multiple features available for selection, the ICC can be used to measure the relative stability of each feature. We show, for 14 distinct data sets (1 synthetic, 8 eye-movement-related, 2 gait-related, and 2 face-recognition-related, and one brain-structure-related), that selecting the most stable features, based on the ICC, resulted in the best biometric performance generally. Analyses based on using only the most stable features produced superior Rank-1-Identification Rate (Rank-1-IR) performance in 12 of 14 databases (p = 0.0065, one-tailed), when compared to other sets of features, including the set of all features. For Equal Error Rate (EER), using a subset of only high-ICC features also produced superior performance in 12 of 14 databases (p = 0. 0065, one-tailed). In general, then, for our databases, prescreening potential biometric features, and choosing only highly reliable features yields better performance than choosing lower ICC features or than choosing all features combined. We also determined that, as the ICC of a group of features increases, the median of the genuine similarity score distribution increases and the spread of this distribution decreases. There was no statistically significant similar relationships for the impostor distributions. We believe that the ICC will find many uses in biometric research. In case of the eye movement-driven biometrics, the use of reliable features, as measured by ICC, allowed to us achieve the authentication performance with EER = 2.01%, which was not possible before.

## Introduction

Suppose that we have multiple testing sessions in which measurements are made. There may be many factors that differ between the two occasions such as temperature, humidity, light intensity, health status, level of fatigue, etc. Also, there will be some degree of measurement error between the two assessments. This means that repeated measurements on the same subject will not be identical. This type of random, unpredictable variation is important to understand. We refer to it as "occasion variance". This model applies to biometrics, because factors listed above potentially might impact biometrics performance. Such a study can be analyzed with a “random-effects” statistical model.

A different type of study, that is more common, but not relevant to biometrics, is a study where some intervention or treatment is applied between measurement sessions. For example, a subject may be put on a weight training program or a diet. Generally, the effect of these treatments or interventions are of great scientific interest. It would not be appropriate in such cases to think of the repeated measurements as randomly varying. Instead we should try to use a statistical technique that addresses the treatment/intervention. This technique would be described as a “fixed effect” statistical model. Such studies are not the subject of this discussion.

For our biometric study, a random sample of subjects is collected. We note that subject 1 may always (systematically) score higher than subject 2. Maybe subject 1 is healthier, or has a clearer cornea, or is more experienced or has better vision than subject 2. This kind of variance is termed “subject variance". And finally, we have variance due to error, which we define as a series of small, unspecified factors, including measurement error. The errors are modelled as statistically independent, having mean 0, and as being drawn from a normal distribution.

We can use a linear statistical model to measure these variances [1]. In this model, each score for each person on each occasion is modelled as the sum of a systematic subject effect, an occasion effect, and random error. If we have an ANOVA table from our experiment, and we follow the method described by Shrout and Fleiss [2], we can calculate all of these variances. Total variance is the sum of subject variance, occasion variance and error variance.

“Occasion-related” changes can occur over a short term (nanoseconds) or over decades. Occasion effects can show no mean change or they can show an increase or a decrease. As discussed below, the biometric community has been focused on one kind of change: "template aging". This is over a long term (e.g., decades), and it is change which carries a negative connotation (e.g., a decrement in performance, a degradation of some body part). However, any change, regardless of whether it is due to an occasion effect (either short-term or long-term), or random error, is a threat to biometric recognition. So, we argue that, to some extent, the focus on "template aging" in biometrics is too specific. Any change is a problem.

At this point, it is helpful to introduce the concept of measurement classes, *inter*class correlation and *intra*class correlation. For this, we cite the introduction to McGraw and Wong [3]:

“To measure the bivariate relation of variables representing different measurement classes, one must use an interclass correlation coefficient, of which there is but one in common use, the Pearson r. Thus, the Pearson r is used for measuring the relation of IQ points (a class of measurement representing aptitude) to grade point averages (a class of measurement representing achievement) or the relation of measurements in the length class (e.g., inches) to measurements in the weight class (e.g., pounds). Such measurements share neither their metric nor variance. But when one is interested in the relationship among variables of a common class, which means variables that share both their metric and variance, intraclass correlation coefficients (ICCs) are alternative statistics for measuring homogeneity, not only for pairs of measurements but for larger sets of measurements as well.” McGraw and Wong, 1996, pg. 30.

Since we are measuring the same subjects on the same measures on two occasions, our data are of the intraclass form. The intraclass correlation coefficient (ICC) measures the temporal persistence from occasion 1 to occasion 2, and can be calculated from the variances mentioned above. In the psychometric community, and the medical research community, the ICC is thought of as a measure of reliability, but in the present context it is reasonable to think of it as measure of “temporal persistence” or “stability”. First introduced by R.A. Fisher 1925 [4], it is the ratio of subject variance to total variance. (Please note that total variance is variance due to subjects plus variance due to occasion plus error variance.) The ICC calculation lumps together change from occasion 1 to occasion 2 and change due to error. It works in the presence of effects that that are short-term or long-term. It works in the presence of decreases and increases. It works in the case of no systematic change. We present the ICC to the biometric community to optimally index the temporal persistence of their biometric features. For biometrics, we want the subjects to vary on our features (large subject variance), and we do not want occasion variance or error variance. We are not studying systematic change from occasion 1 to occasion 2, although we are planning to conduct this analysis in the scope of our future work. There are different statistical procedures that can be employed to determine the statistical significance and effect size of systematic effects. We are not studying the amount of error variance. We are trying to identify human subjects and we do not want our features to change over time, *at all*, *for any reason*.

In this work, we introduce the ICC as an index of temporal persistence or stability. (We are aware that some biometric researchers have used the ICC [5], although, as far as we can tell, not to represent temporal persistence.) We show how it is calculated and interpreted, and we then study the effects of sorting features in terms of ICC ("temporal persistence" or "stability") on biometric performance in 14 databases. We hypothesize that the ICC has very wide application to many biometric situations because temporal persistence is a requirement for a well performing biometrics trait. This index of stability, or temporal persistence works best for data that are on an interval or ratio scale, and are reasonably normally distributed. Other statistically based measures of stability that apply to continuous non-normal data, ordinal data or nominal type measures are available but are not discussed in the present report, but will be a part of our future work. To the extent that a biometric system depends on multiple measures with these characteristics, the ICC of each feature indicates how stable that feature is in absolute terms and in relation to other features. In this current work, we have used the ICC for feature selection, and assessed the impact of its use on biometric performance.

The ICC ranges from 0.0 to 1.0. Cichetti, and Sparrow [6, 7] have suggested that ICC levels be interpreted by the following rules of thumb: ICC > = 0.75: “Excellent”, ICC > = 0.60, and < 0.75: “Good”, ICC > = 0.40, and < 0.60: “Fair”, and ICC < 0.40: “Poor”. Not only does the ICC provide information within a study of the relative temporal persistence of a feature, it provides an estimate of the absolute level of stability of a feature, which can be compared across feature types, e.g., from anatomical characteristics to personality traits. We expect that features that are based on physiological (e.g., electrocardiogram or electroencephalogram) or behavioral modalities (e.g., speech recognition, gait, or eye-movements) will have lower stability generally than features based on physical characteristics (fingerprints, iris scan, face). The precise degree of this difference in stability can be quantified with the ICC.

### Prior work

We are not aware of any prior biometric study which employed a test-retest paradigm, and evaluated the ICC as an index of temporal persistence of potential features. We have found 1 paper with a title (“ICC statistic as criterion for classification and feature selection”) [8] but the description provided in the paper did not make it possible to understand how ICC was used for feature selection. We found one paper that employed the ICC in the context of feature selection for face recognition biometrics [5]. These authors did not employ the ICC to assess temporal persistence. The data structure in that paper differed from the data structure in the present case. In that paper, there were multiple face images for each subject. The ICC was used to find features which were consistent across all the faces for everyone.

**Fig 1:**
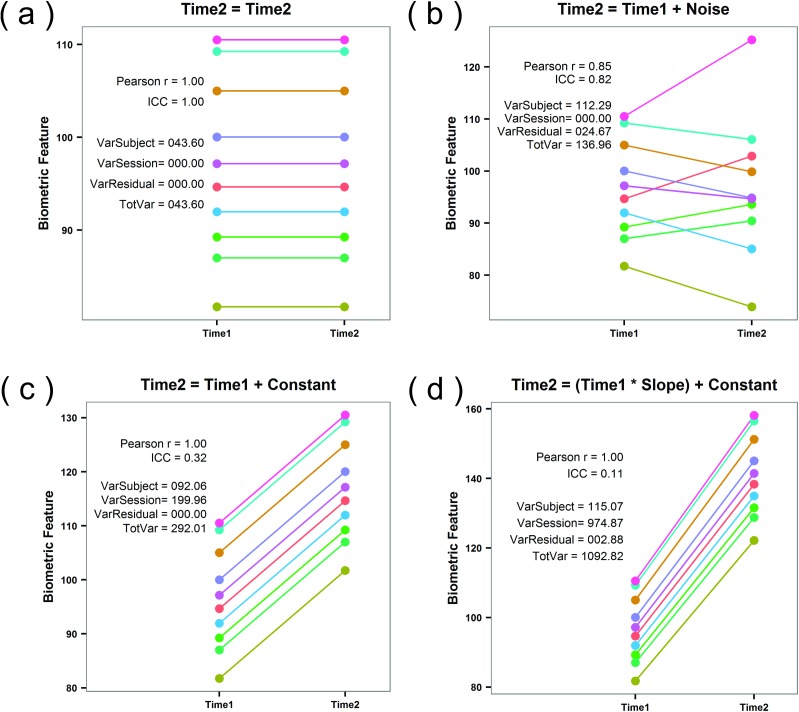
Features of the Pearson r correlation coefficient and the ICC. (a) For illustration purposes only, we have drawn a random sample of 10 “subjects” from a population with mean = 100 and SD = 10. These are the Time1 data for all plots. In this case, we have set Time2 = Time1, and both the Pearson r and the ICC are equal to 1.0. (b) In this figure, the Time2 data are equal to the Time1 data but with added noise drawn from a population with mean = 0 and SD = 10. Both the Pearson r and the ICC decline somewhat, which is appropriate because the relationship between Time1 and Time2 is now weaker. (c) For the Time2 data for this plot, we did not add noise, but merely added 20.0 to the Time1 data. This is essentially representing a “session” or “occasion” effect. In this case, the ICC has declined substantially but the Pearson r is still at a perfect 1.0. (d) In this case, we did not add noise to Time1 data, but performed a linear transformation of Time1 data to get Time2 data. The ICC has dropped precipitously whereas the Pearson r is still at 1.0. This is what we mean when we say the that the Pearson r is invariant to linear transformation. The 4 variances below the ICC are used to calculate the ICC, as explained in the S1 Document.

We have found one paper that employed the Pearson’s r to assess repeatability [9], and another paper that used the t-value for a regression of time 2 onto time 1 [10]. The Pearson’s r is an *inter*class correlation coefficient, whereas the ICC is an *intra*class correlation coefficient. The former is designed for measures that are from different classes (different variables with different variances). As illustrated in Fig 1, Pearson r is invariant to linear transformation of the time 2 data with respect to the time 1 data. In contrast, the ICC is a measure of absolute, not relative, agreement, and is appropriate when all the data are from the same class (same variables with similar variance). Anything which prevents one from getting the same score will reduce the ICC but may not affect Pearson’s r (Fig 1). The same can be said of a t-value from a linear regression, i.e., this is also invariant to linear transformations. Another study created their own “stability index” [11], which is based on the average absolute deviation from either the mode or the mean (for discrete versus continuous measures, respectively). Why these researchers chose to invent their own stability index, rather than use the already well established Kendall Coefficient of Concordance (first described in 1939 [12], for continuous, not normally distributed data) or the ICC (first described in 1925, [4], for continuous, normally distributed data) is not clear. For assessments on a nominal scale, prior researchers have employed percentage agreement between time1, and time2 [13]. As noted by the authors as well as Bartko [14] and others, this procedure ignores chance agreements, and is considered a less suitable measure than Kappa [15]. One study employed an interesting meta-analytic technique to assess the reliability of nominal assessments by fingerprint experts [16].

The effect of time on biometric systems has been addressed by the biometric community, with a focus on “template aging”. For reviews, see [17, 18]. These approaches study the effect of time on biometric results, such as an ROC curve, EER, Rank-1Rank-1-IR or false acceptance rate. The findings in the template aging field are inherently multivariate, since biometric results depend on the use of all features at once [19–34]. The aim of this literature is not to produce an index of temporal persistence of a single feature, but rather to assess the global effect of time on biometric performance, using many features at once. Since the literature on template aging does not produce a competitive metric of temporal stability of a single feature, as we do here, we do not think this literature is relevant to the present report.

This paper introduces the notion of temporal persistence in biometrics, enumerates it using the intraclass correlation coefficient, and evaluates it on several databases of various modalities to demonstrate validity, and efficacy of this notion.

## Materials and methods

### Databases

#### Overview

We report on 14 databases. All data sets are briefly outlined here, and explained in detail below. There is 1 synthetic database, 8 eye-movement-related databases (Table 1), 2 gait-related databases (Gait-Area Based Metrics (GABM), and Gait-Zernike Velocity Moment-Based (GZVMB)), two FACE Recognition databases (FACE1 and FACE2), and one brain-structure-related database (BRAIN). All the short-term eye-movement databases are based on the same subjects, using the same eye-movement recordings, during the same task. In this case, the test-retest interval is on the order of 19 minutes (within this report, repeated testing periods on the same subjects within a day are referred to as “sessions”). All the long-term eye-movement databases are based on the same subjects, using the same eye-movement recordings, during the same task. In this case, the test-retest interval is on the order of 11.1 months (range: 7.8 to 13.0 months). The eye-movement data were collected as part of a larger database collected at Texas State University for the NSF CAREER grant awarded to Dr. Komogortsev. The eye-movement databases differ, however, in the sense that different, but not necessarily mutually independent, sets of features were extracted from eye-movement signals (details below). The subjects were reading a poem for up to 60 seconds. SBA is a new database, based on a new comprehensive feature set which is described briefly below, but in more detail in a corresponding report [35]. CEM-12-DB is based on the approach described in [36]. OPC-18 is based on the features described in [37]. CEM-14 is based on the approach described in [38]. Both gait-related databases employ the Southampton Large Population Gait database of gait-related images, and videos [39]. These databases are comprised of over 100 subjects tested on many sessions. Generally, the gait assessments analyzed herein were all collected during the same day, often as little as 1 minute apart. In the present analysis, we report only on the Session 1 vs Session 2 comparison. GABM employs area-based metrics [40]. GZVMB is based on Zernike velocity moments extracted from these images [41]. Note that these gait-related databases, and analyses were chosen to illustrate the importance of the assessment of temporal persistence. They do not represent the best performance achievable with gait-related databases. The same statement is true for the face databases. The brain-related database consists of brain volume measures of several brain structures in 40 subjects measured twice.

**Table 1:** Full names and abbreviations for eye movement databases.

Number	Full Name	Abbreviation
1	Statistical Biometric Approach-Short-Term	SBA-ST
	Statistical Biometric Approach-Long-Term	SBA-LT
3	Complex Eye Movement-12-Features-Distribution-Based-Short-Term	CEM-12-DB-ST
4	Complex Eye Movement-12-Features-Distribution-Based-LongTerm	CEM-12-DB-LT
5	Oculomotor Plant Characteristics-18 Features—Short Term	OPC-18-ST
6	Oculomotor Plant Characteristics-18 Features—Long Term	OPC-18-LT
7	Complex Eye Movement-14-Features-Short-Term	CEM-14-ST
8	Complex Eye Movement-14-Features-Long-Term	CEM-14-LT

The eye movement data collection was approved by the IRB of Texas State University, San Marcos, TX and was conducted per the principles expressed in the Declaration of Helsinki. The gait-related databases were created prior to the initiation of an IRB at the University of Southampton. The gait data was collected under the auspices of the DARPA Human ID at a Distance program. We do not know under what conditions the faces we used for FACE1 or FACE2 were collected. We do know that the images are in the public domain and that no identifying personal information was included with the image databases. The BRAIN data were collected with IRB approval at each of the clinical sites were subjects were evaluated.

#### Synthetic database

**Fig 2:**
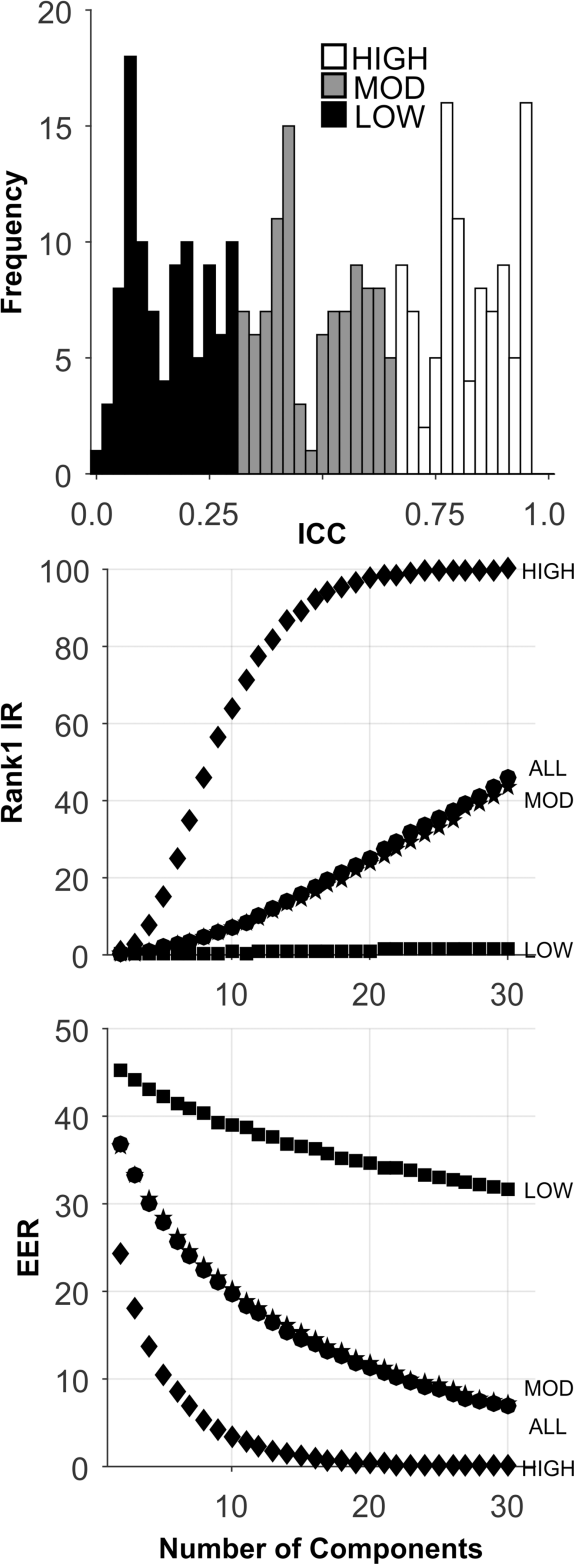
Analysis of the Synthetic database. Top plot: ICC Histogram. Low ICC features (black bars), moderate ICC features (gray bars), high ICC features (white bars). Middle plot presents the Rank-1-IR results as a function of the number of PCA components. Lower plot presents the EER results. Each symbol represents the median over 100 random training-testing sets.

We created a synthetic database comprised of 1000 subjects, tested twice (2 sessions) with 300 features (Table 2). For each feature, subjects were assigned a number from 1 to 1000, randomly sampled without replacement, from a vector containing the integers in that range. The subject was assigned that number for both her session 1 data and her session 2 data. Then random noise, sampled from a normal distribution with varying standard deviations (SD), was added to the score for each subject and each session. Different random noise values were assigned to session 1 and session 2. For each feature a unique random noise distribution, characterized by a unique standard deviation of the noise, was employed. The mean of each feature tended to be near 500 (500.4, SD = 11.3, min = 453.9, max = 557.4). The average SD was also near 500 (501.1, SD = 225.0, min = 291.3, max = 1161.6). The maximum intercorrelation between features (absolute value) was 0.105, and the median intercorrelation (absolute value) was 0.017. The 300 features varied in relative temporal persistence (ICC ranged from near 0.0 to near 1.0, see Fig 2). There were 297 normal, unique features (Table 2). Four ICC sets were constructed: (1) “HIGH” consisted of 99 features with the highest ICCs, (2) “MOD” consisted of 99 features with moderate ICCs, (3) “LOW” consisted of 99 features with low ICCs, and (4) “ALL” included all 297 features. See Table 2 for cut-points for the 3 ICC databases. The mean ICC for all databases is presented in Table 2.

**Table 2:** Description of databases.

Analysis	Reference	Number Of Subjects	Test-Retest Interval	Number of Original Features	Number of ICC Sets	Final Number of Features in each ICC Set^a^	Mean ICC	Cut Points
Synthetic	N/A	1000	N/A	300	4	L: 99, M: 99, H: 99, A: 297	0.49	0.31,0.66
SBA-ST	N/A	298	Median 19 min	1,027	4	L: 122, M: 122, H: 122, A: 366	0.53	0.41, 0.71
SBA-LT	N/A	68	Median 11.1 mon	1,027	4	L: 33, M: 33, H: 33, A: 33	0.38	0.24, 0.51
CEM-12-DB-ST	[36]	298	Median 19 min	84	3	L: 21, H: 21, A: 42	0.54	N/A
CEM-12-DB-LT	[36]	68	Median 11.1 mon	84	3	L: 25, H: 25, A: 18	0.45	N/A
OPC-18-ST	[37]	298	Median 19 min	18	3	L: 8, H: 8, A: 16	0.44	N/A
OPC-18-LT	[37]	68	Median 11.1 mon	18	3	L: 9, H: 8, A: 17	0.46	N/A
CEM-14-ST	[38]	298	Median 19 min	14	3	L: 4, H: 4, A: 8	0.80	N/A
CEM-14-LT	[38]	68	Median 11.1 mon	14	3	L: 6, H: 6, A: 12	0.59	N/A
GABM	[40]	112	Within Same Day	74	3	L: 34, H: 34, A: 55	0.70	N/A
GZVMB	[41]	107	Within Same Day	31	3	L: 11, H: 11, A: 22	0.88	N/A
FACE1	N/A	40	Within Same Day	1,024	4	L: 19, M: 19, H: 19, A: 19	0.59	0.49,0.612
FACE2	N/A	152	Within Same Day	2,250	4	L: 75, M: 75, H: 75, A: 74	0.77	0.76,0.84
BRAIN	[42]	40	1 Week	37	3	L: 18, H: 18, A: 19	0.80	N/A


^a^ L is for the LOW ICC Set, M is for the MOD ICC Set, H is for the HIGH ICC Set, and A is for the ALL ICC Set.

Initially, the size of sets 1 (L), 2 (M) and 3 (H) were equal. The number of features in this column have gone through screening for normality and redundancy. Also, when necessary, features were removed to achieve positive definiteness of the correlation matrix for the database.

#### SBA-ST database

This analysis, and database have not been described in print before, so a more extensive discussion of the database will be presented for this database only.

The analysis presented here is the result of many important prior steps, including subject recruitment, eye-movement recording, eye-movement classification, feature extraction, assessment of normality of feature distributions, reliability assessment, feature redundancy elimination, and biometric assessment. These phases are being described in detail in separate publications in preparation. Therefore, in this manuscript, the prior steps will be described only briefly.

The subjects were all undergraduate college students at Texas State University (N = 333, N Female = 157, N Male = 178, mean age = 21.8, range = 18 to 46 yrs., SBA-Short-Term).

On each subject visit, subjects were studied twice (Sessions 1, and 2), approximately 20 min apart (SBA-Short-Term). Each Session included 7 different sets of visual stimuli, only 1 of which (Poetry Reading) is relevant to the present report. For each instance of the Poetry Reading task, a different pair of quatrains from the famous nonsense poem, “Hunting for a Snark”, written by Lewis Carroll (written from 1874–1876), were displayed. Subjects were asked to read the poem portion silently. They were given 60 seconds to read this poem passage. Session 1 to Session 2 (task-to-task) time intervals ranged from 13 min to 42 min (mean: 19.5; SD: 4.2, (SBA-Short-Term)).

The subjects were seated 55 cm in front of a computer monitor with their heads resting on a chin/head rest. The monitor subtended +/- 23.3 degrees of visual angle in the horizontal direction, 11.7 degrees to the top, and 18.5 degrees to the bottom. The EyeLink 1000 (SR Research Ltd., Ottawa, ON, Canada) was employed for eye-movement recording. It is a video-oculography system, which records both horizontal, and vertical eye movements. The sampling rate for our data was 1000 Hz. In the present study, only left eye movements were collected. The device has reported accuracy of 0.25–0.5 deg. For empirical accuracy estimates and other basic statistics regarding the eye movement recordings, see the supplementary document: “**S2 Document**–Basic Statistics and Accuracy Estimates”. For further technical specifications of the EyeLink 1000, see [43].

In the present study, all eye movements were analyzed off-line. We began our classification of the eye movements using the method described by Nystrőm, and Holmqvist [44], using Matlab code kindly made available by Dr. Nystrőm. Over the course of many months, we modified this code so extensively to enhance its performance for our data that what we have ended up with is an effectively new eye-movement classification scheme, based on the general approach outlined in [44]. We are preparing a manuscript for publication describing the many changes we made to this algorithm, and assessing the performance of the new algorithm in comparison to the original code.

Briefly, the algorithm classifies eye-movement signals into fixation periods, saccades, post-saccadic oscillations, and noise. It relies most heavily on the velocity trace, and largely ignores position and acceleration for classification purposes. We only analyzed that portion of the signal where, according to our analysis, subjects were actually reading the poem. Most of the subjects took the entire 60 sec. to read the poem, but many subjects finished early. Ten subjects did not have both Session 1, and Session 2 recordings, and twenty-five subjects were dropped from the study due to low recording quality. This left 298 subjects (Table 2). Our overall philosophy regarding feature extraction was to extract every conceivable, quantifiable, and objective feature we could think of. The idea was that this very large set of features would be winnowed down to a reasonable number of features in several steps. We did not try to guess a priori which features would be useful, and which would not be useful, but rather determined the usefulness of the features empirically. Many features were obviously redundant (e.g., means, and medians), but this redundancy was removed in a later stage. For most measures, features were separately extracted for the horizontal component, the vertical component, and a radial component. For a more detailed discussion of the features employed, see Rigas et al [35].

Most of the features were based on distributions of measures for each event type (fixation, saccade, and post-saccadic oscillation), and for each such measure we extracted the mean, median, standard deviation, interquartile range, skewness, and kurtosis.For fixations, we measured duration, rate (per second), fixation drift, velocity noise, shape in the position channel, shape in the velocity channel, and shape in the acceleration channel. For saccades, we measured duration, rate, shape in the positon channel, shape in the velocity channel, shape in the acceleration channel, peak velocity, peak acceleration, saccade trajectory curvature in the 2-dimensional plane (horizontal, and vertical), as well as main sequence relationships. For post-saccadic oscillations, we measured size, percentage of saccades with post-saccadic oscillations, shape in the positon channel, and shape in the velocity channel, shape in the acceleration channel, peak velocity, and peak acceleration. We also evaluated reading speed, number of small saccades (< 8 deg) to the right (presumably word-to-word saccades), number of small saccades to the left (presumably refixation saccades), and number of line-returning saccades. Finally, we also measured types, and character of artifacts, as well as pupil size. A manuscript providing a detailed presentation of the construction of the various features is available [35]. As a result of feature extraction, we had 1027 potential eye-movement features for each subject, for each Session. After removal of features which were not normal, and could not be transformed into normal, and after removal of redundant features, the features were divided up into 4 ICC sets (LOW, MOD, HIGH, ALL), See Table 2 for sample sizes, test-retest interval, number of features, number of ICC sets, ICC set sizes, mean ICC, and relevant cut-points for ICC sets.

#### SBA-LT database

The data are of exactly the same form as the SBA-Short-Term database, but the subjects are a subset of 78 subjects retested at approximately 11.1 months. Ten of the subjects had excessively noisy recordings, and so 68 subjects were analyzed. See Table 2 for sample sizes, test-retest interval, number of features, number of ICC sets, ICC set sizes, mean ICC, and relevant cut-points for ICC.

#### CEM-12-DB database

Short-Term: This database employs 12 simple eye-movement features (Table 3), 6 of which are simple main sequence measures relevant to saccades. Each of these features has a distribution within each subject, representing the values for each fixation, and each saccade. In the original paper [36], the best results were obtained when the entire distributions were compared. In the present application, we extracted the mean, median, SD, 25th percentile, 75th percentile, skewness, and kurtosis from each of these 12 distributions for each subject, for a total of 84 features. See Table 2 for sample sizes, test-retest interval, number of features, number of ICC sets, ICC set sizes, and mean ICC.

**Table 3:** List of features for CEM-12-DB, OPC-18 and CEM-14 databases.

Number	CEM-12-DB	OPC-18	CEM-14
**1**	Start time (fixation)	Series Elasticity (AG)	Number of Fixations
2	Duration (fixation)	Series Elasticity (ANT)	Mean Fixation Duration
3	Horizontal centroid (fixation)	Length-Tension Relationship (AG)	Mean Radial Saccade Amplitude
4	Vertical centroid (fixation)	Length-Tension Relationship (ANT)	Mean Horizontal Saccade Amplitude
5	Start time (saccade)	Force-Velocity Relationship (AG)	Mean Vertical Saccade Amplitude
6	Duration (saccade)	Force-Velocity Relationship (ANT)	Mean Radial Saccade Velocity
7	Horizontal amplitude (saccade)	Passive Viscosity	Mean Radial Saccade Peak Velocity
8	Vertical amplitude (saccade)	Tension Slope (AG)	Slope of the Amplitude/Duration Relationship (saccades)
9	Horizontal mean velocity (saccade)	Tension Slope (ANT)	Slope of the Main Sequence Relationship
10	Vertical mean velocity (saccade)	Inertial Mass	Velocity Waveform Indicator
11	Horizontal peak velocity (saccade)	Activation Time (AG)	Scanpath Length
12	Vertical peak velocity (saccade)	Activation Time (ANT)	Scanpath Convex Hull Area
13		Deactivation Time (AG)	Regions of Interest
14		Deactivation Time (ANT)	Inflexion Count
15		Tension Intercept	
16		Neural Pulse (AG)	
17		Neural Pulse (ANT)	
18		Neural Pulse Width	

Long-Term: The data are of exactly the same form as the CEM-12-DB-Short-Term database, but only in the long-term subset of subjects (N = 68) retested at approximately 11.1 months. See Table 2 for sample sizes, test-retest interval, number of features, number of ICC sets, ICC set sizes, and mean ICC.

#### OPC-18 database

Short-Term: Dr. Komogortsev and colleagues have developed an analysis of saccade data, by which estimates can be made of 18 oculomotor plant characteristics (OPC-18) representing the internal anatomical structure of the eye, including the extraocular muscles, the eye globe, surrounding tissues, and the dynamics of the neuronal control signal (Table 3) [37]. We extracted these 18 features from the same poetry reading database described above. Thus, there were 298 subjects, with two sessions each, with 18 features per subject per session. These features were divided into three sets, a HIGH ICC set, a LOW ICC set (above, and below the median), and a third set with all the 18 features (“All”). See Table 2 for sample sizes, test-retest interval, number of features, number of ICC sets, ICC set sizes, and mean ICC.

Long-Term: The data are of exactly the same form as the OPC-18-Short-Term database, but only in the long-term subset (N = 68) of subjects retested at approximately 11.1 months. See Table 2 for sample sizes, test-retest interval, number of features, number of ICC sets, ICC set sizes, and mean ICC.

#### CEM-14 database

Short-Term: This database is based on horizontal eye movements only and employs 14 fairly simple eye-movement features listed in Table 3, and described in full in [38]. After normalization testing, and redundant feature removal, we had 10 features to test. See Table 2 for sample sizes, test-retest interval, number of features, number of ICC sets, ICC set sizes, and mean ICC.

Long-Term: The data are of exactly the same form as the CEM-14-Short-Term database, but only in the long-term subset (N = 68) of subjects retested at approximately 11.1 months. See Table 2 for sample sizes, test-retest interval, number of features, number of ICC sets, ICC set sizes, and mean ICC.

#### Gait databases

Both gait databases are based on the Southampton Large Population Gait database of gait-related images, and videos [39]. These databases are comprised of over 100 subjects tested on many sessions. Sessions can be as little as 1 minute apart. Both approaches start with a series of image frames while a subject walked. Both began by extracting binary silhouettes of the walking human. At this point the databases differed.

For GABM, the silhouette extraction used chroma-key subtraction in conjunction with a connected components algorithm. The silhouettes were resized to 64 x 64 pixels. A series of image masks (*m* masks), like a mask for a horizontal band near the subject’s waist, or the upper half of the silhouette, were applied to each subject silhouette, for each consecutive frame, and a time series for each mask type for each subject for each session was produced. At this stage, each subject was characterized by the time series of *m* masks. A cubic spline curve was fitted for the whole gait cycle, and 30 evenly spaced samples were taken from the whole curve, giving a single vector for each area mask used. These multiple vectors, for each 1 to *m* mask, were reduced to a fewer dimensions, using canonical analysis. For each subject, the first feature was the first value in the final single vector, the second feature was the second value in this vector, and so on.

For GZVMB, the silhouette extraction used both luminance values and edges to determine the background and foreground information. Silhouette extraction was based on scene variance and standard deviation. The silhouettes were then resized to 128 x 160 pixels. Image moments were calculated on these silhouettes [45]. Zernike moments are one particular type of moment, described in Prokop and Reeves [45]. Typically, these moments are computed on silhouettes from static images. Shutler and Nixon [41] have extended the application of Zernike moments to the velocity of moving silhouettes. Our data consists of 31 such moments measured for 107 subjects, tested twice on the same day.

#### FACE1 database

For this database, we used the face images in the Olivetti Research Limited (ORL) database of faces. The database consists of 400 images, 10 each for 40 different subjects (6 females). The subjects were either Olivetti employees or Cambridge University students. The age of the subjects ranges from 18 to 81, with most between the ages of 20–35. We used the ORL_32x32 database, available online at ORL_32x32 (AT&T Laboratories, Cambridge). There are 10 different images of each of 40 distinct subjects. For some subjects, the images were taken at different times, varying the lighting, facial expressions (open / closed eyes, smiling / not smiling) and facial details (glasses / no glasses). All the images were taken against a dark homogeneous background with the subjects in an upright, frontal position (with tolerance for some side movement). We analyzed only the first and second session for each subject. The images are grey scale and 32 x 32 pixels in size.

For analysis, we first zscore-transformed the intensity in all the images. Then we created an average face from all the subjects in the first session and subtracted this average face from all images from both sessions. For biometric identification, we initially considered all 1024 pixel intensities as 1024 distinct features. For session 1, we would have an input array of 40 rows (subjects) by 1024 pixels. We next determined which pixels were normally distributed, or could be transformed to normality. Those that were not normal and could not be transformed to normality, we dropped. Next, we determined the ICC of each of the 1024 features. We then checked for pairs of pixels that were correlated greater than 0.95 (absolute value), and removed the pair member that had the lower reliability. At this stage, the database was split into three subsets, based on ICC. We created a low ICC database, a moderate ICC database, a high ICC database, and an all ICC database, using the cut points listed in Table 2. We then submitted each database to a PCA analysis. For each of these databases, the PCA analysis using all features would not run properly, because the correlation matrices were not positive definite. This was due to the presence of too high intercorrelations between features. So, for each database, we found the most highly intercorrelated pairs of features, and removed the less stable member of the pair, until the correlation matrices were positive definite. We did this iteratively, so that as few features as possible would have to be removed from each database. These databases were assessed for biometric performance.

#### FACE2 database

For this database, we used the face images available online at faces94. The images are made available by Dr Libor Spacek, from the University of Essex (United Kingdom). The data consist of 153 subjects, tested on multiple sessions. The images are colored (24-bit, RGB) and are 180 x 200 pixels. We analyzed only the first and second session for each subject. During image acquisition, the subjects sat at fixed distance from the camera and were asked to speak, whilst a sequence of photographs was taken. The speech was used to introduce facial expression variation. The background is plain green, with minor variations in head turn, tilt and slant. All images were acquired using constant lighting, with only minor changes in the position of the face in the image. There was considerable variation from session 1 to session 2 in facial expressions. Hair style was constant.

The images were transformed to grayscale, and resized to 50 x 45 pixels. The raw images were very similar from session 1 to session 2, and did not represent a challenge to biometric identification. Therefore, we degraded the session 2 images. First, we added noise to the session 2 images, by adding a random normal deviate with a mean of 0 and an SD of 0.1 to each pixel. Then we rotated the session 2 images, from -10 degrees to 10 degrees, randomly, in steps of 1 degree. As above, we created an average image across all subjects from session 1 and subtracted this average image from all images in the study. For biometric identification, we initially considered all 2250 pixel intensities as distinct features.

All the subsequent steps for this analysis were identical to those for the FACE1 database. This included checking for normality and using transformations to normality, looking for redundant features, creating ICC sets, and iteratively removing highly intercorrelated variables to achieve a positive-definite correlation matrix, suitable for subsequent PCA.

#### Brain database

The brain-structure database (BRAIN) consist of the volumes of 37 brain structures, for 40 subjects, measured twice, 1 week apart. The list of brain structures is provided in Table 4. The MRI data for this database was collected as part of a research project entitled: “Establishing Moderators and Biosignatures of Antidepressant Response for Clinical Care for Depression” (EMBARC). The database is described in Iscan et al [42]. It was made available to us via a request to the publication committee of EMBARC.

**Table 4:** Brain structures measured in the BRAIN database.

Brain
3rd Ventricle
4th Ventricle
Brain Stem
Corpus Callosum—Anterior
Corpus Callosum—Central
Corpus Callosum–Mid-Anterior
Corpus Callosum–Mid-Posterior
Corpus Callosum—Posterior
Cerebral Spinal Fluid
Optic Chiasm
Accumbens area (L, R)
Amygdala (L, R)
Caudate (L, R)
Cerebellum Cortex (L, R)
Cerebellum White Matter (L, R)
choroid plexus (L, R)
Hippocampus (L, R)
Inferior Lateral Ventricle (L, R)
Lateral Ventricle (L, R)
Pallidum (L, R)
Putamen (L, R)
Thalamus Proper (L, R)
Ventral Diencephalon (L, R)

High quality T1-weighted brain MRI scans were collected on both occasions, and analyzed with FreeSurfer, a software package which performs several functions, including the automatic measurement of the volume of various brain structures and regions [46] (Freesurfer). In the first step, the brain volumes were assessed for normality or transformed into normal. One of the volumes measured is the volume of the entire brain. It is likely that many of the remaining brain structures would be correlated with this measure, since, all other things being equal, subjects with larger brains will also have larger volumes of regional brain structures. So, the 36 remaining measures were regressed onto the brain volume measure, and only the residuals of these regressions were saved as features. All of the remaining steps in the analysis followed the steps outlined below for all databases.

### Data analysis

**Fig 3:**
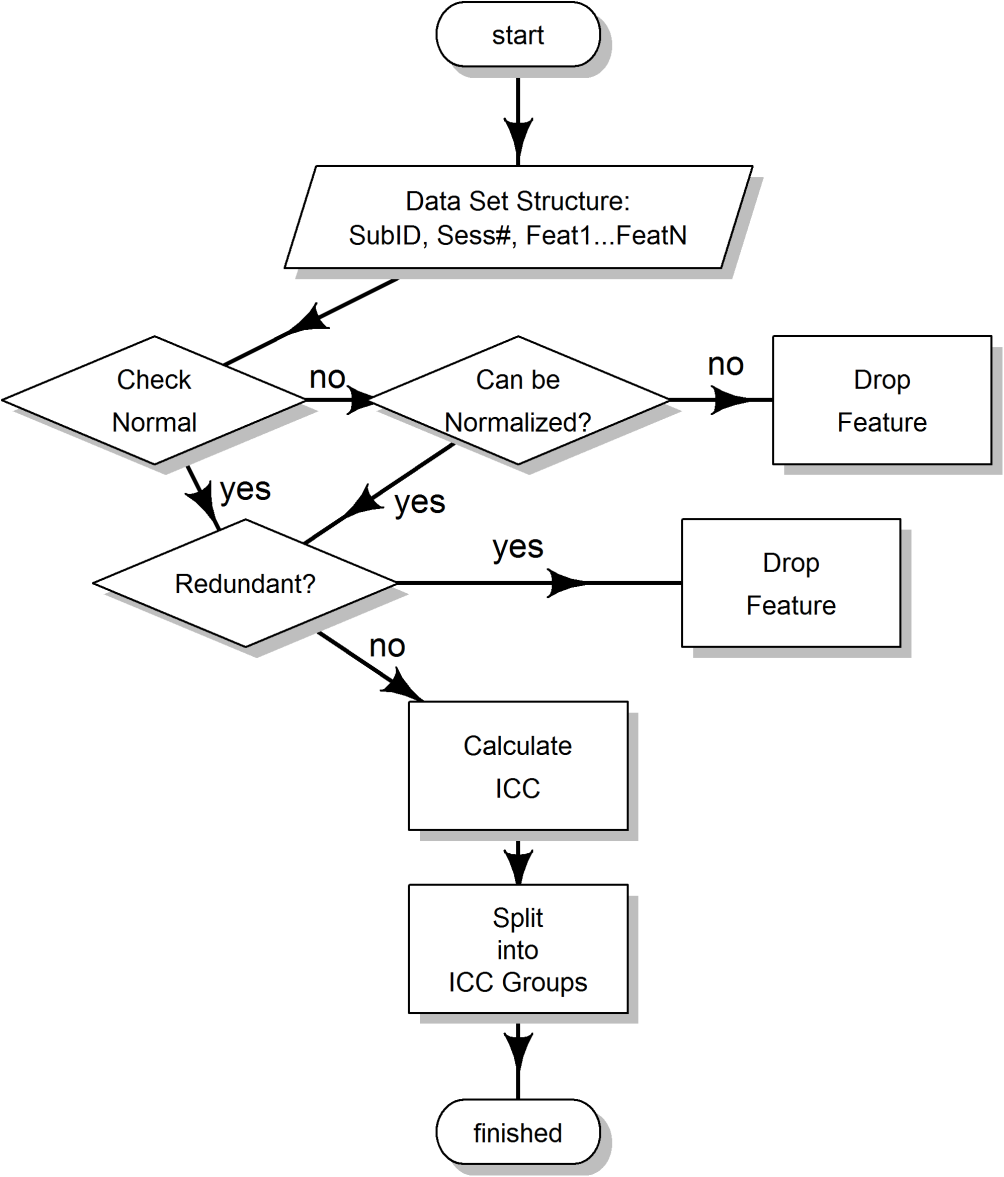
Flowchart of the data analysis. See text for details.

Fig 3 presents general overview of our analysis.

#### Initial feature set reduction

Our first step for all databases was to determine which features were normally distributed or could be transformed to normality using variable transformations. This was done using all data from all subjects from the first session. We wanted to assess the reliability of each measure using the ICC, which requires that the data be normally distributed. We assessed normality using the Pearson Chi-Square test. For measures that were not normally distributed, standard transformations were applied to the data (reciprocal, log, square root, cube root, reflected, logit, arcsine, winsorization [47, 48]). Measures which were not normally distributed, and could not be transformed into normal, were dropped from further analysis.

#### Removal of redundant features

Several databases contained redundant features. For example, some features were based on either the mean or the median of the same distribution, or the standard deviation (SD) or the interquartile range (IQR) of the same distribution. We did not need two estimates of the central tendency of eye-movement feature distributions (mean, and median) so the less reliable (lowest ICC) measure was dropped from further analysis. Similarly, we did not need two measures of variance [interquartile range (IQR), and standard deviation (SD)] so the less reliable was dropped. We also intercorrelated every feature with every other feature, and found those pairs of features that were intercorrelated (Pearson’s r) greater than 0.95 (absolute value). We considered such pairs of features effectively redundant. The lower reliability feature from each pair was dropped from further analysis.

#### Calculation of the intraclass correlation coefficient

See S1 Document for a detailed discussion of the calculation of the ICC. Briefly, for each database, for each feature, we have *n* subjects and *k* = 2 occasions. The score for *j*^th^ occasion (*j* = 1:k) of the *i*^th^ subject number (i = 1:*n*), X_*ij*_, is represented under the two-way random effects model as:

$$X_{ij}=Subject\_Effect_{i}+Occasion\_Effect_{j}+Error_{ij},$$

Subject_Effect is assumed to be normally distributed with mean 0 and variance σ^2^_S_; Occasion_Effect is the random effect of occasion *j*, and Error_*ij*_ is random error, also assumed to be normally distributed with mean 0 and variance σ^2^_e_. Under this model, it is assumed that all variables are mutually independent. The intraclass correlation coefficient (ICC) is given by:

$$ICC=\frac{{\hat{\sigma }}_{S}^{2}}{{\hat{\sigma }}_{X}^{2}}=Subject\_Variance/Total\_Variance=\frac{n*(MeanSquareSubject-MeanSquareError)}{(n*MeanSquareSubject)+(k*MeanSquareOccasion)+(nk-n-k)\;*MeanSquareError}$$

With all the mean squares obtained from a fixed-effect two-way ANOVA [2].

#### Splitting the features into ICC sets

When there were many features (> 200, Synthetic, SBA) we split the features into 3 equally sized ICC sets (High, Moderate, Low). When there were fewer features (CEM-12-DB, OPC-18, CEM-14, GABM, and GZVMB) the features were split into 2 equally sized ICC sets (High, Low). In addition, for all databases, an “ALL” ICC set, containing all features, regardless of ICC, was also considered.

#### Assessing the positive-definiteness of ICC data sets

During the assessment of biometric performance (see below), the data were separated into training, and testing sets, each containing N/2 subjects. PCA analysis was performed on each training set. PCA requires correlation matrices as input that are positive-definite (all eigenvalues > 0.0). Sometimes, the PCA algorithm would fail due to the non-positive definiteness of the relevant correlation matrix. In this case, we searched for the most highly intercorrelated pairs of features, and removed the member of a pair with a lower ICC value. We removed as few features as necessary to get the PCA to work without error.

### Assessing biometric performance

A set of features, typically N components from a PCA, were used for biometric identification. The distance between subjects on these features in N-dimensional space was computed, using cosine distance. The cosine distance was scaled from 0 to 1, and reflected (1-scaled distance) to form similarity scores. It should be noted that several other types of distances were tested (Euclidean, City-block, Spearman, Mahalanobis), and provided competitive performance. Since analyses based on the Cosine distance performed marginally better than the other distance metrics, the results we present here are based on that metric.

Biometric performance was assessed in terms of Rank-1 Identification Rate (Rank-1-IR), and equal error rate (EER).

Equal Error Rate (EER): The EER is a measure of the verification accuracy of a biometric system. A genuine score is defined as the score from the comparison of the biometric samples coming from the same identity. An impostor score is defined as the score from the comparison of biometric samples coming from different identities. By defining an acceptance threshold (η) we can compute the False Rejection Rate (FRR) as the percentage of the genuine scores that fall under the threshold η, and the False Acceptance Rate (FAR) as the percentage of the impostor scores that are over η. True Positive Rate (TPR) can be defined as the percentage of genuine scores that are over the threshold η, with TPR = 1—FRR. By changing the acceptance threshold, we can construct a Receiver Operating Characteristic (ROC) curve, and calculate the EER as the point of operation where the FRR equals the FAR.

Rank-1 Identification Rate (Rank-1-IR): The Rank-k Identification Rate (Rank-k-IR) is a measure of biometric identification performance which shows the percentage of genuine scores that can be found within the k top places of a ranked list. A Cumulative Match Characteristic (CMC) curve shows the change of the identification rate as a function of the used rank k. The Rank-1-IR is defined as the percent of biometric samples with a correct match in the first place of the ranked list.

Biometric performance was assessed at the level of an ICC set [HIGH, MOD (Synthetic, SBA, FACE1, FACE2), LOW, ALL]. Each ICC set consisted of k features for N subjects for 2 sessions. For each ICC set, the following procedures were followed: The set of N subjects from Session 1 was split into a training set (N/2 subjects), and a test set (N/2 subjects) using random sampling, without replacement. A principal component analysis (PCA) was conducted for dimension reduction, extracting from 2 to p components, where p is determined by the data set, and ranged from 3 for the OPC-18-Short-Term data to 30 for the Synthetic Data Base. Components were extracted until a peak Rank-1-IR performance was achieved. Using the component structure from the training set, we created component scores for the test sample, both Session 1, and Session 2. These testing data sets were submitted to a biometric performance analysis treating Session 1 data as the gallery, and Session 2 data as the probe. We present the median Rank-1-IR, and the EER over the 100 random test samples for from 2 to p components. For CEM-14 only, since there were so few features, no PCA was conducted. All features were entered the biometric assessment algorithm directly.

### Analyses across databases

We performed 3 analyses which combined data from all the databases. When we say all, we do not include the CEM-14 databases (CEM-14-ST, CEM-14-LT) because these databases had very few features and PCA could not be conducted. For each database type, there were either two (CEM-12-DB-ST, CEM-12-DB-LT, OPC-ST, OPC-LT, GAIT1, GAIT2, BRAIN) or three (Synthetic, SBA-ST, SBA-LT, FACE1, FACE2) ICC subsets. These 29 ICC subsets form the basis of all of the analyses described in this section. The first step was to determine the median ICC for each of these 29 ICC subsets. The median ICC was the independent variable for all the analyses described in this section.

#### ICC vs Rank-1-IR or EER

For these analyses, we determined both the Rank-1-IR and the EER for each entire ICC subset. We then regressed Rank-1-IR or EER on the median ICC. For this, we employed a Mixed Model with “dataset” modelled as a random factor. We show the relationships for these two measures (Rank-1_IR and EER) in scatterplots, and include statistical information (F-value, df, p-value, r^2^) on each plot.

#### Median similarity scores

For these analyses, we determined the median of similarity scores for the genuine distribution and for the impostor distribution. We then regressed the median similarity scores (genuine or impostor) on to median ICC. For this, we employed a Mixed Model with “dataset” modelled as a random factor. We show the relationships between median similarity score (genuine or impostor) to median ICC in scatterplots, and include statistical information (F-value, df, p-value, r^2^) on each plot. Next, we tested for a difference between the first slope (relationship between median genuine similarity scores and median ICC) and the second slope (median impostor similarity scores and median ICC) using a mixed model ANOVA. The dependent variable was the similarity score, the “genuine vs impostor” factor was treated as a repeated measure fixed effect, and median ICC was treated as a covariate. We tested for a main effect of the “genuine vs impostor” distinction, the fixed effect of the covariate and also for the covariate by “genuine vs impostor” factor interaction. If the interaction was statistically significant, we concluded that the slopes mentioned above were statistically significantly different (i.e., we can reject the null hypothesis that the slopes were equal).

#### IQR similarity scores

For these analyses, we determined the IQR of similarity scores for the genuine distribution and for the impostor distribution. We then regressed the IQR similarity scores (genuine or impostor) on to median ICC. For this, we employed a Mixed Model with “dataset” modelled as a random factor. We show the relationships between IQR similarity score (genuine or impostor) to median ICC in scatterplots, and include statistical information (F-value, df, p-value, r^2^) on each plot. We used the same strategy mentioned above for comparing the two slopes.

## Results

### Synthetic database

The analyses for this database are presented in Fig 2 and Table 5. By design, there was a wide range of ICC values for these 297 features (Fig 2, Top). For all sets, performance improved as the number of components extracted increased to 30. For the HIGH ICC set, Rank-1-IR performance was perfect (100.0, Fig 2, Middle, and Table 5). For EER, the HIGH ICC set also performed perfectly (EER = 0.0, Fig 2, Bottom, Table 5). The performance of the HIGH ICC set was dramatically better than the performance of the other sets, including the ALL set, which contained more features by a factor of 3.

**Table 5:** Biometric performance summary analysis.

Database Name	Rank 1—IR			EER		
	Best Set	Highest Rank 1 –IR	Number of Components	Best Set	Lowest EER	Number of Components
Synthetic	HIGH	100.0	30	HIGH	0.00	30
SBA-ST	HIGH	90.6	22	HIGH	2.69	19
SBA-LT	HIGH	70.6	13	HIGH	10.16	10
CEM-12-DB-ST	HIGH	48.3	14	HIGH	11.41	12
CEM-12-DB-LT	HIGH	44.1	13	HIGH	20.59	10
OPC-18-ST	HIGH	9.4	3	HIGH	21.48	3
OPC-18-LT	HIGH	23.5	8	ALL	25.67	3
CEM-14-ST	ALL	33.6	N/A *	HIGH	15.44	N/A *
CEM-14-LT	HIGH	50.0	N/A *	HIGH	15.86	N/A *
GMB	HIGH	80.4	15	HIGH	5.36	11
GZVMB	ALL	83.0	10	ALL	5.66	10
FACE1	HIGH	77.5	13	HIGH	9.74	10
FACE2	HIGH	97.4	12	HIGH	1.54	12
BRAIN	HIGH	95.0	6	HIGH	5.00	3

*For CEM-14 no PCA was performed.

All features were entered into each analysis.

### SBA database

**Fig 4:**
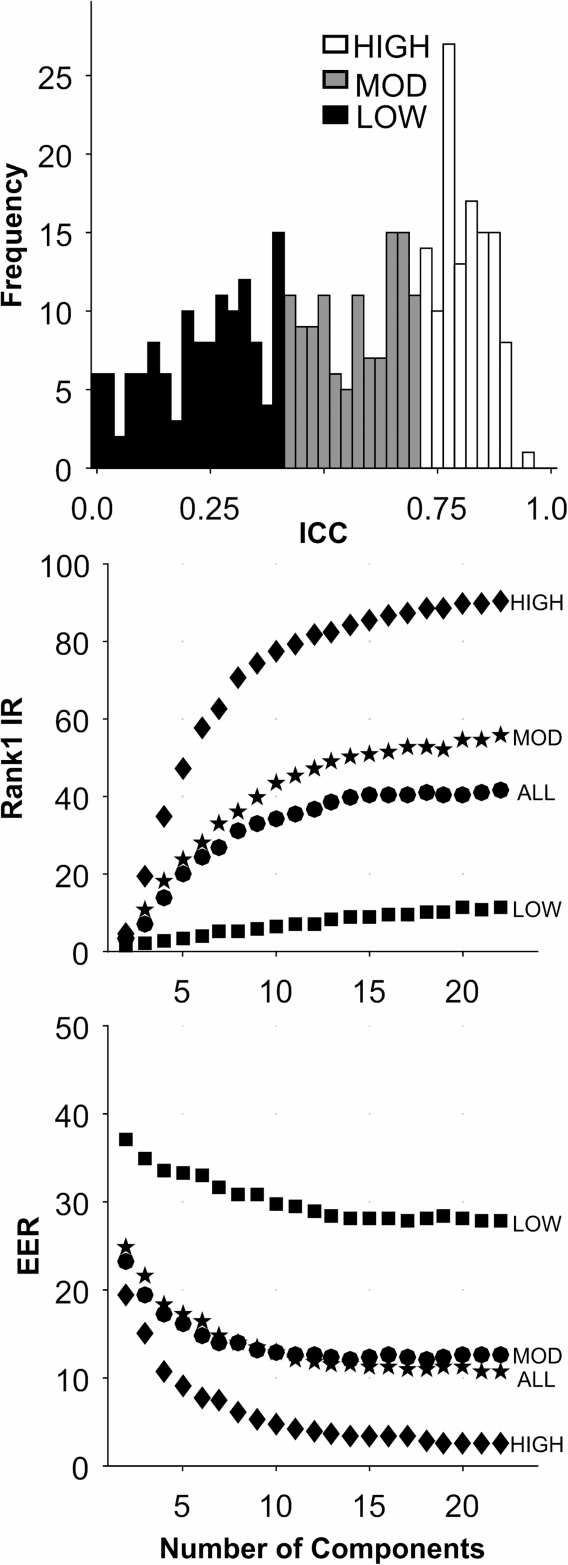
Analysis of the SBA-Short-Term database. See caption for Fig 2.

Short-Term: The analyses for this database are presented in Fig 4, and Table 5. There was a large range of ICCs for these 366 features (Fig 4, Top). For all sets, performance generally improved as the number of components extracted increased to 22. For the HIGH ICC set, Rank-1-IR performance was very good (Fig 4, Middle, and Table 5). For EER, the HIGH ICC set also performed very well (Fig 4, Bottom, Table 5). The performance of the HIGH ICC set was dramatically better than the performance of the other sets, including the ALL set, which contained more features by a factor of 3.

As noted in Table 5, the best performance was found for the High ICC group (90.60) for Rank-1-Identification Rate (22 components), and 2.68 EER (19 components). This occurred with ICC > 0.71. With further experimentation, we could do even better by changing the way we performed the PCA. First, before we performed the PCA, we searched for “natural components”, by finding features weakly correlated with the set of all other features [49]. We found 7 raw features that, at most, had a correlation with a single other feature of 0.7. These natural components were not entered into the PCA. Also, we thresholded the ICCs at 0.75 rather than 0.71. After re-running the analysis with 17 PCA components and 7 “natural components”, we achieved a Rank-1-Identification Rate of 91.95 with an EER of 2.01 with 17 PCA components and 7 natural components. With 19 PCA components and 7 natural components, we achieved a Rank-1-Identification Rate of 92.62 with an EER of 2.43.

**Fig 5:**
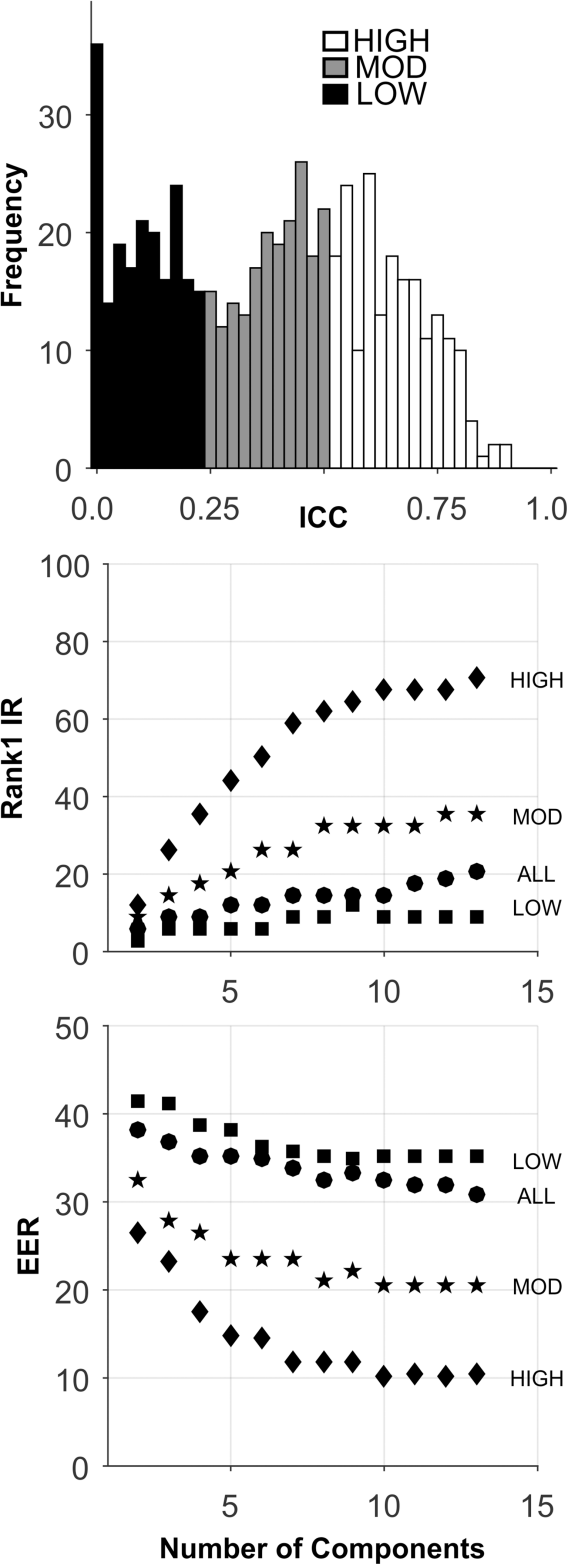
Analysis of the SBA-Long-Term database. See caption for Fig 2.

Long-Term: The analyses for this database are presented in Fig 5, and Table 5. There is a large range of ICCs for these features (Fig 5, Top). Note the decline in mean ICC compared to SBA-Short-Term (0.53 to 0.38, Table 2). We expected ICC to decrease as a function of the length of the test-retest interval. For all sets, Rank-1-IR performance generally improved as the number of components extracted increased to 13. For the HIGH ICC set, Rank-1-IR performance was good (Fig 5, Middle, and Table 5), although not as good as for the SBA-Short-Term database (Rank 1-IR: 90.6 to 70.6). For EER, the HIGH ICC set also performed better than any other database (with 10 components), although the performance was not excellent (Fig 5, Bottom, Table 5). The performance of the HIGH ICC set was dramatically better than the performance of the other sets, including the ALL set, which contained more features by a factor of 3.

### CEM-12-DB database

**Fig 6:**
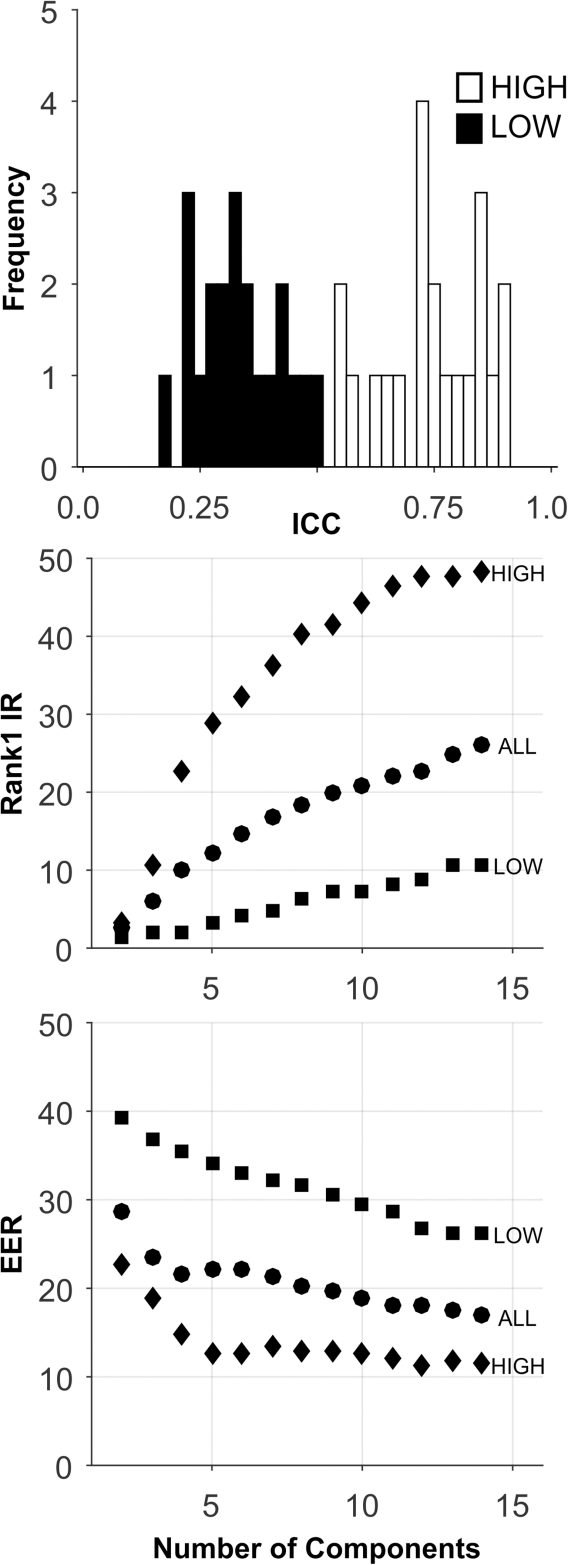
Analysis of the CEM-12-DB-Short-Term database. See caption for Fig 3.

Short-Term: The analyses for this database are presented in Fig 6, and Table 5. There was a large range of ICCs (Fig 6, Top). For all sets, for Rank-1-IR, performance generally improved as the number of components extracted increased to 14. For the HIGH ICC set, Rank-1-IR performance was not impressive (Fig 6, Middle, and Table 5), but was much higher for the HIGH ICC set than for the LOW set, and the ALL set, even though the ALL set contained twice as many features as the HIGH ICC set, including the HIGH ICC features. For EER, the HIGH ICC set also performed better than the other sets (Fig 6, Bottom, Table 5), and achieved its best performance at 12 components, but the level of performance was not very good.

**Fig 7:**
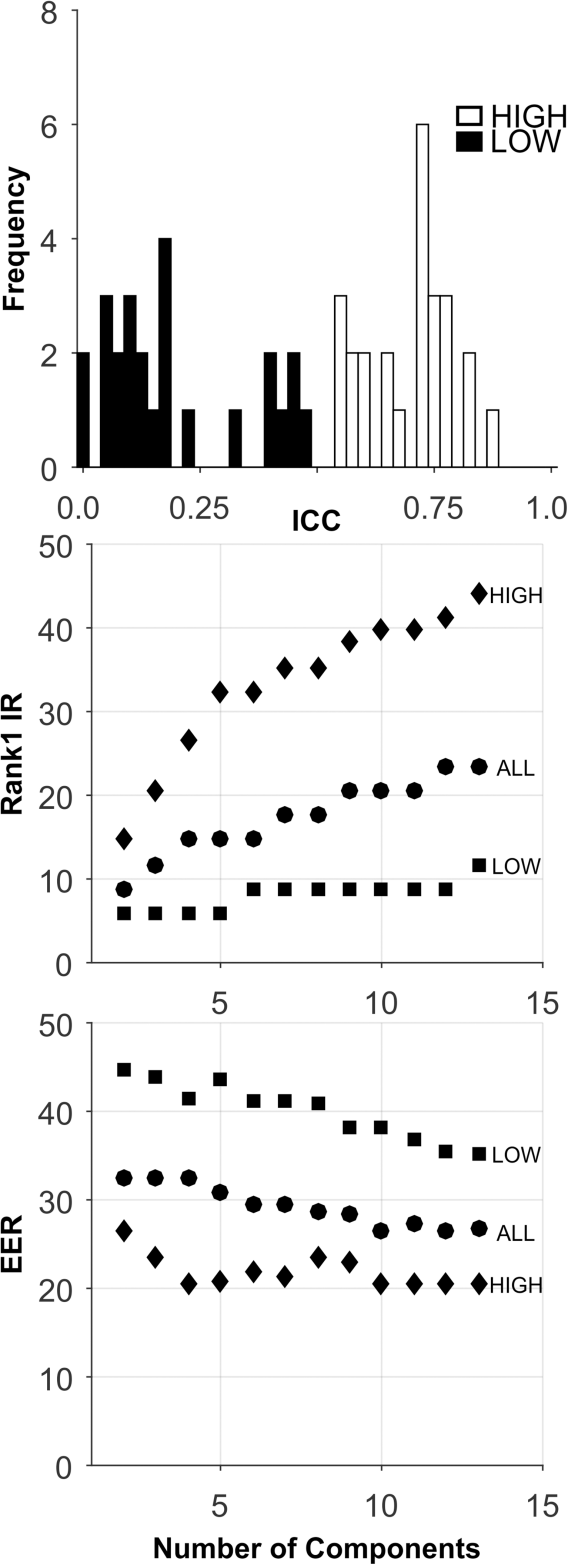
Analysis of the CEM-14-Long-Term database. See caption for Fig 2.

Long-Term: The analyses for this database are presented in Fig 7, and Table 5. There was a drop in the mean ICC from the CEM-14-ST (Mean ICC: 0.54 to 0.45). Despite this, the performance of all the ICC sets is like the performance for the CEM-14-Short-Term database. Clearly the HIGH ICC database performed best for both Rank-1-IR, and EER, and clearly outperformed the “ALL” database.

### OPC-18 database

**Fig 8:**
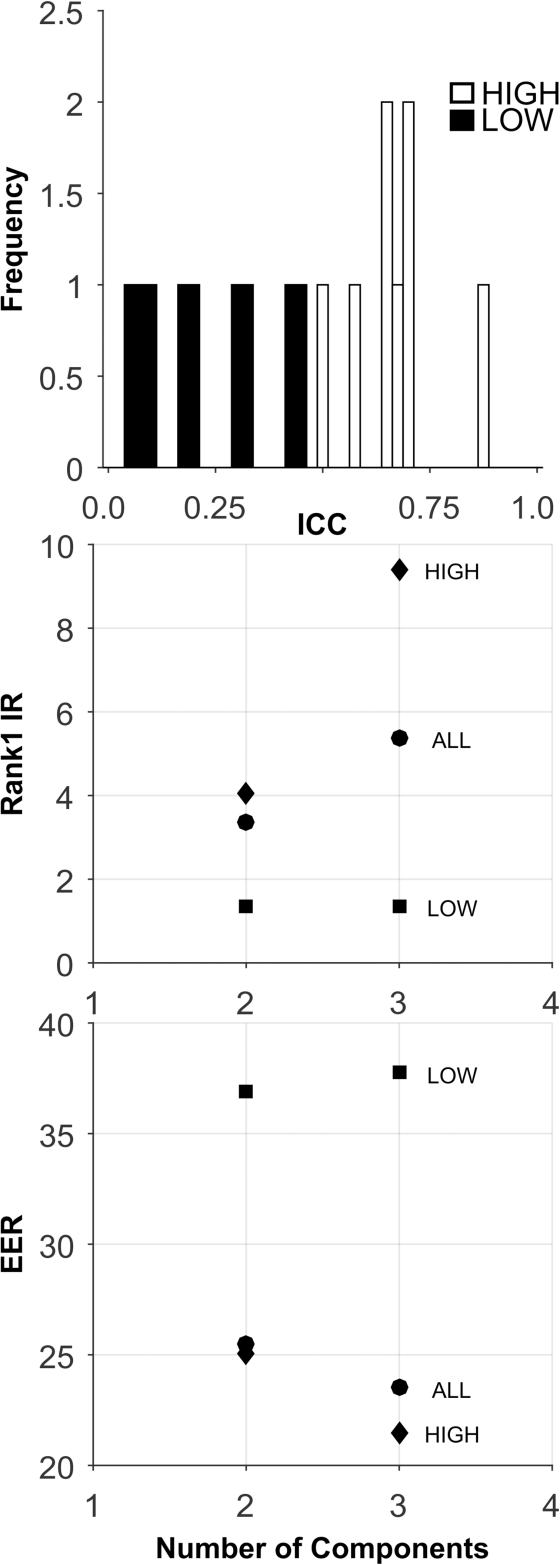
Analysis of the OPC-18-Short-Term database. See caption for Fig 2.

Short-Term: The analyses for this database are presented in Fig 8, and Table 5. There are only 18 features for this database. There was some variability in the ICC range, but generally, the ICCs are poor for these features (Fig 8, Top, Table 5). For the HIGH ICC set, Rank-1-IR performance was very poor (Fig 8, Middle, and Table 5), but was higher for the HIGH ICC set than for the ALL set, and especially the LOW ICC set, even though the ALL set contained a twice as many features as the HIGH ICC set, including the HIGH ICC features. For EER, the HIGH ICC set was narrowly better than the ALL data (Fig 8, Bottom, Table 5). Nonetheless, EER performance with this database was quite poor.

**Fig 9:**
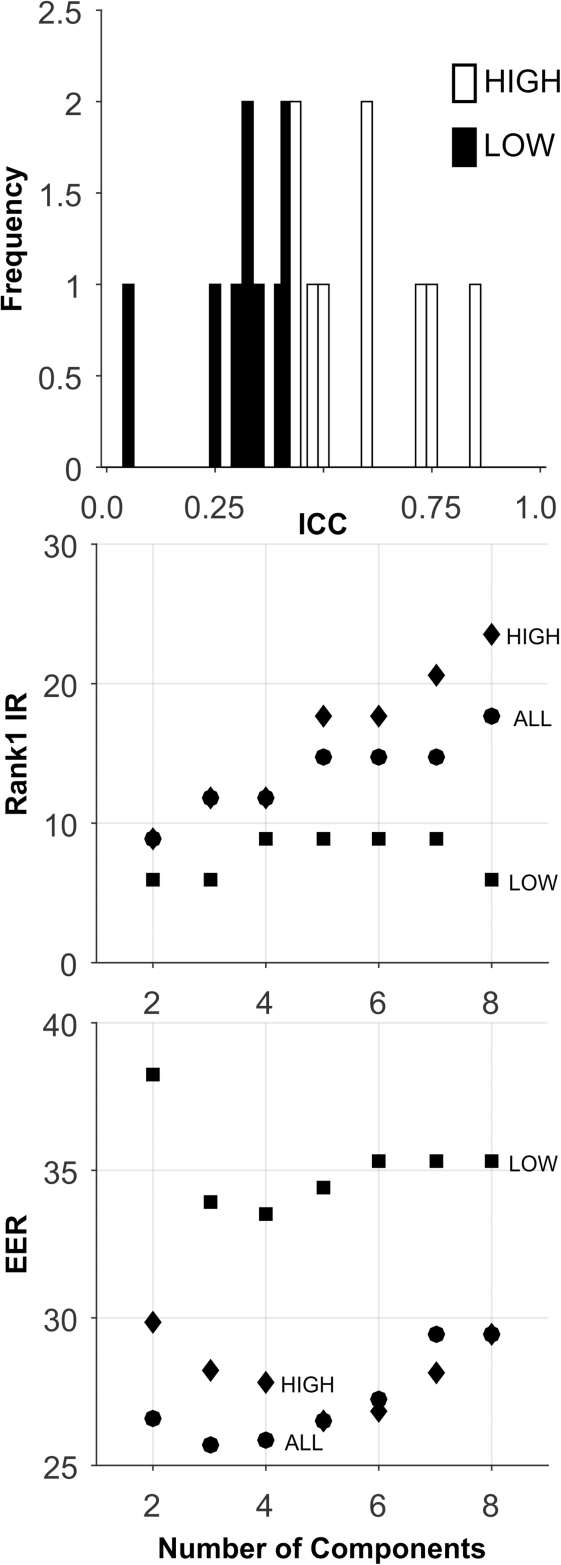
Analysis of the OPC-18-Long-Term database. See caption for Fig 2.

Long-Term: The analyses for this database are presented in Fig 9, and Table 5. The ICCs for this database are not very different from the ICCs for the OPC-18-Short-Term database (mean ICCs: 0.46 vs 0.44, Table 2). The HIGH ICC set outperformed the other sets for Rank-1-IR, but the ALL ICC database performed the best for EER. Overall the performance was poor.

### CEM-14 database

**Fig 10:**
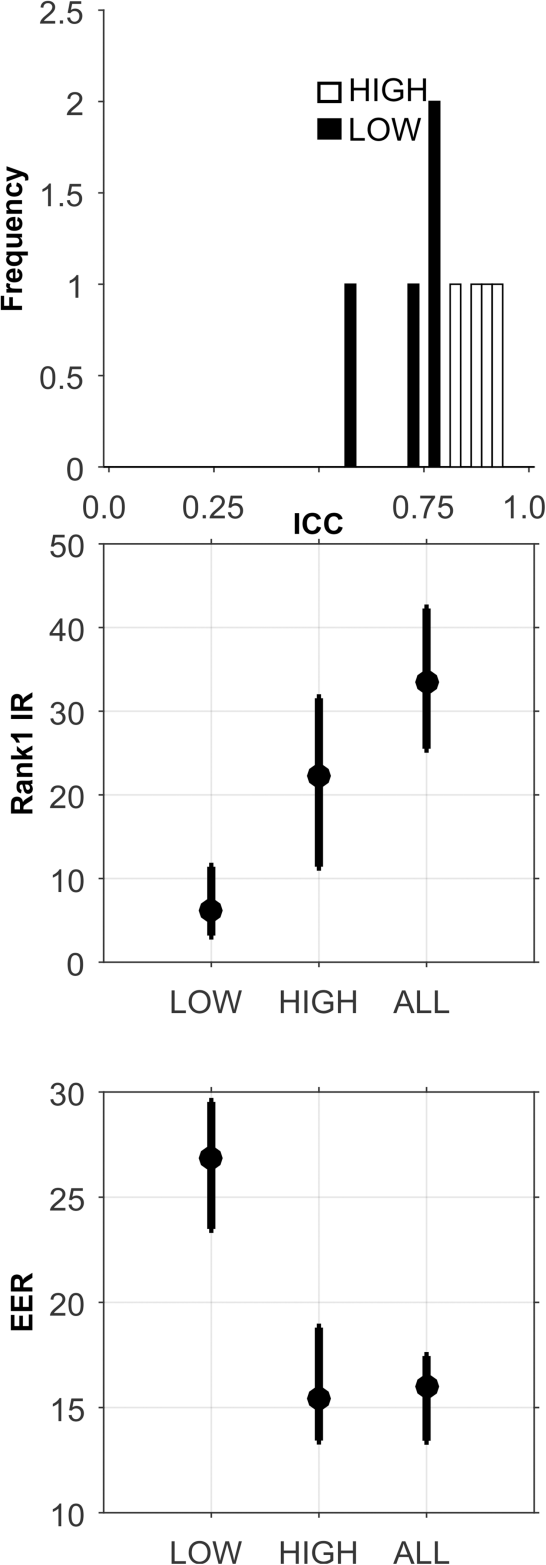
Analysis of the CEM-14-Short-Term database. PCA was not performed, due to the small number of features. All features were entered directly into the biometric assessment algorithm. Plotted are means (dots) and minimum and maximum value error bars based on 100 training and testing sample sets.

Short-Term: The analyses for this database are presented in Fig 10, and Table 5. There was a very small number of features in these ICC sets (Table 2). The ICCs were generally high (Fig 10, Top). With so few features, PCA was not performed for this database. For Rank-1-IR, the best performance was for the ALL ICC set (Fig 10, Middle, and Bottom, and Table 5). However, even this “best performance” was quite poor. For EER, the best performance was for the HIGH set, but the level of performance was still quite poor.

**Fig 11:**
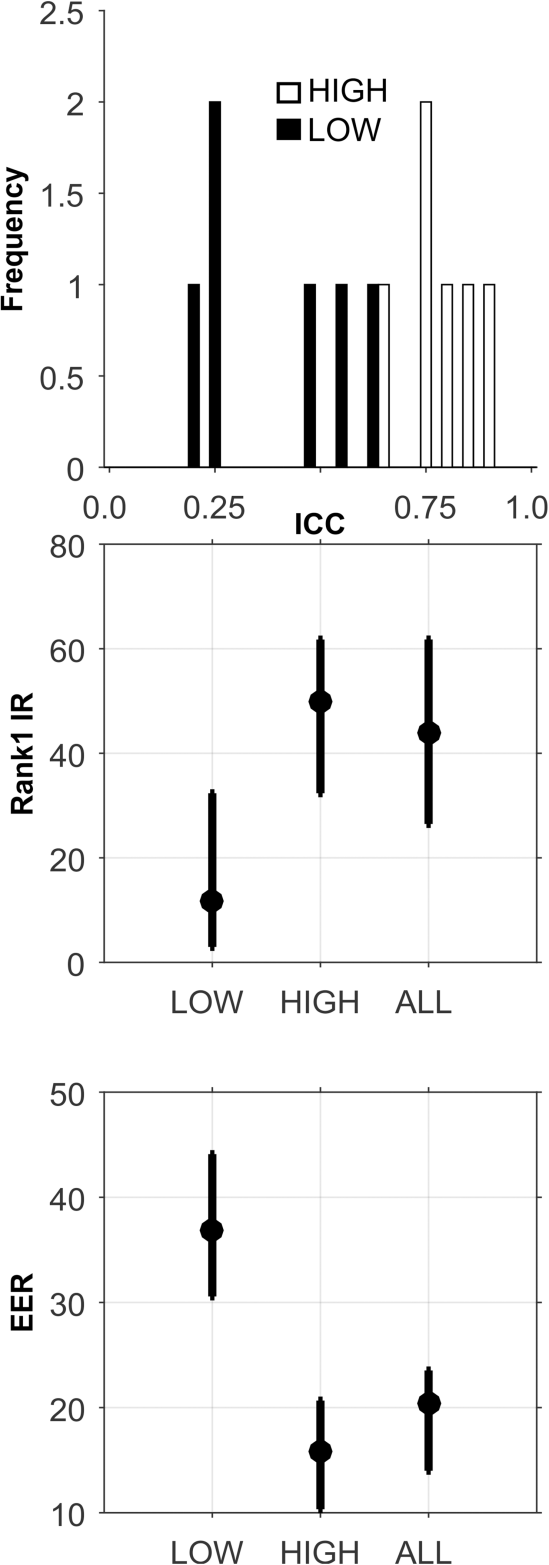
Analysis of the CEM-14-Long-Term database. See caption for Fig 10.

Long-Term: The analyses for this database are presented in Fig 11, and Table 5. The mean ICC dropped substantially from CEM-14-ST (mean ICC from 0.80 to 0.59, Table 2). Rank-1-IR performance was much better for this long-term version of the data, but was still poor. The HIGH ICC set performed better for both Rank-1-IR and EER.

### GABM database

**Fig 12:**
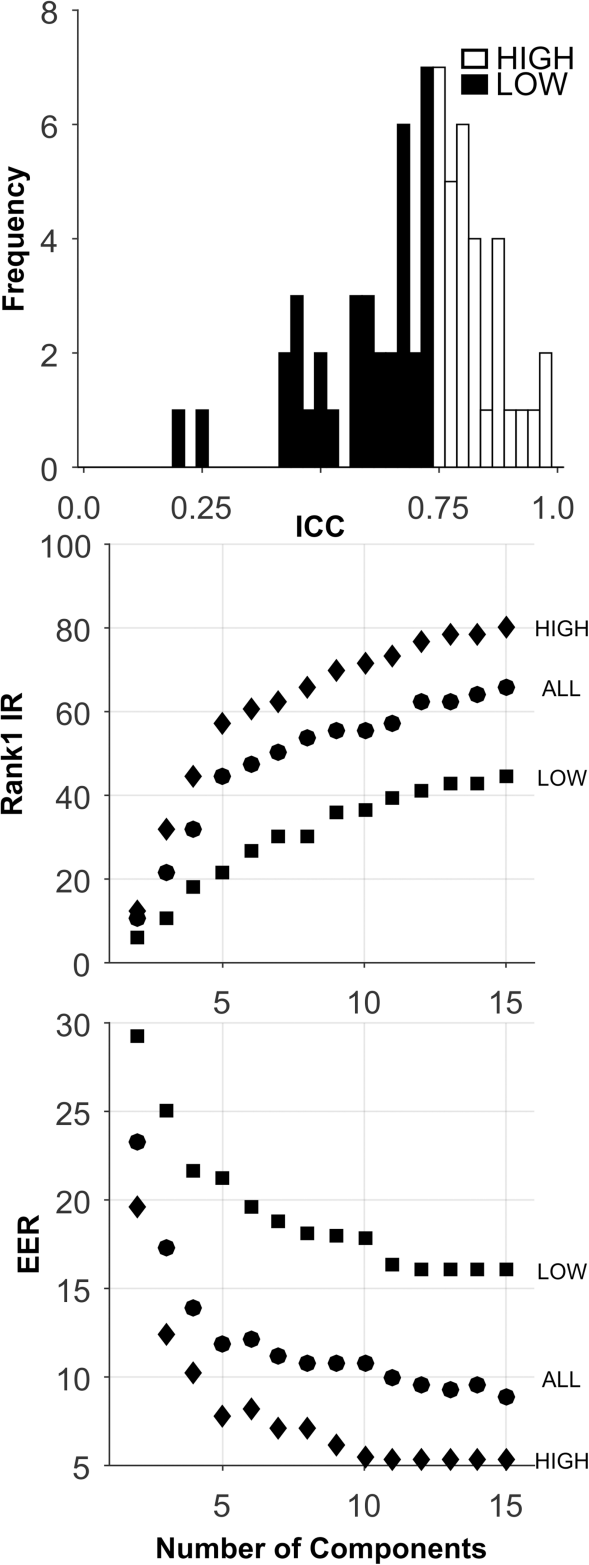
Analysis of the GABM database. See caption for Fig 2.

The analyses for this database are presented in Fig 12, and Table 5. There were 74 original features for this database (Table 2), and the ICCs were generally high (Fig 12, Top). For all sets, for Rank-1-IR, performance generally improved as the number of components extracted increased to 15. The HIGH ICC set performed substantially better than the other ICC sets for both Rank-1-IR (Fig 12, Middle), and EER (Fig 12, Bottom). Overall, the performance was reasonably good for this database.

### GZVMB database

**Fig 13:**
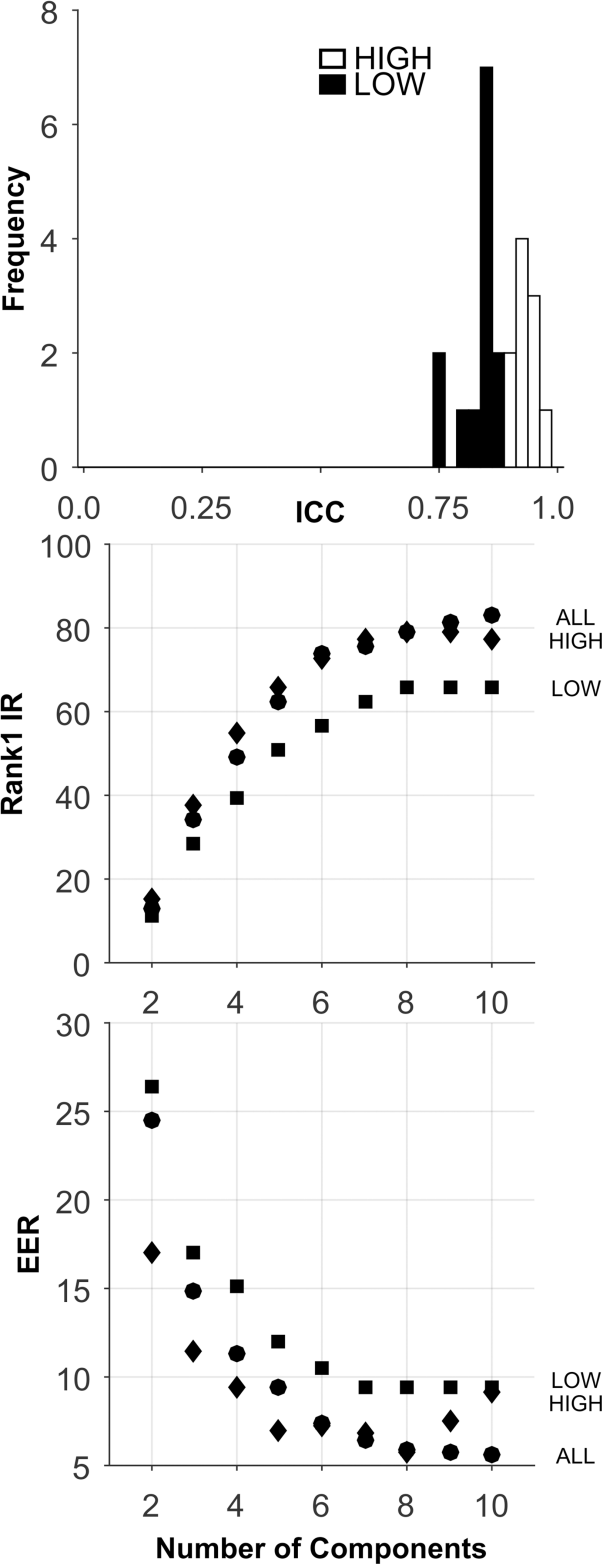
Analysis of the GZVMB database. See caption for Fig 2.

The analyses for this database are presented in Fig 13, and Table 5. There were 31 features for this database (Table 2), and the ICCs were all in the excellent range. This was a very reliable set of features. The ALL ICC set achieved superior Rank-1-IR peak performance at 10 components (Fig 13, Middle). For EER, the best performance was for the also for the ALL ICC set, (Fig 13, Bottom, Table 5). Overall, the performance was reasonably good for this database.

### FACE1 database

**Fig 14:**
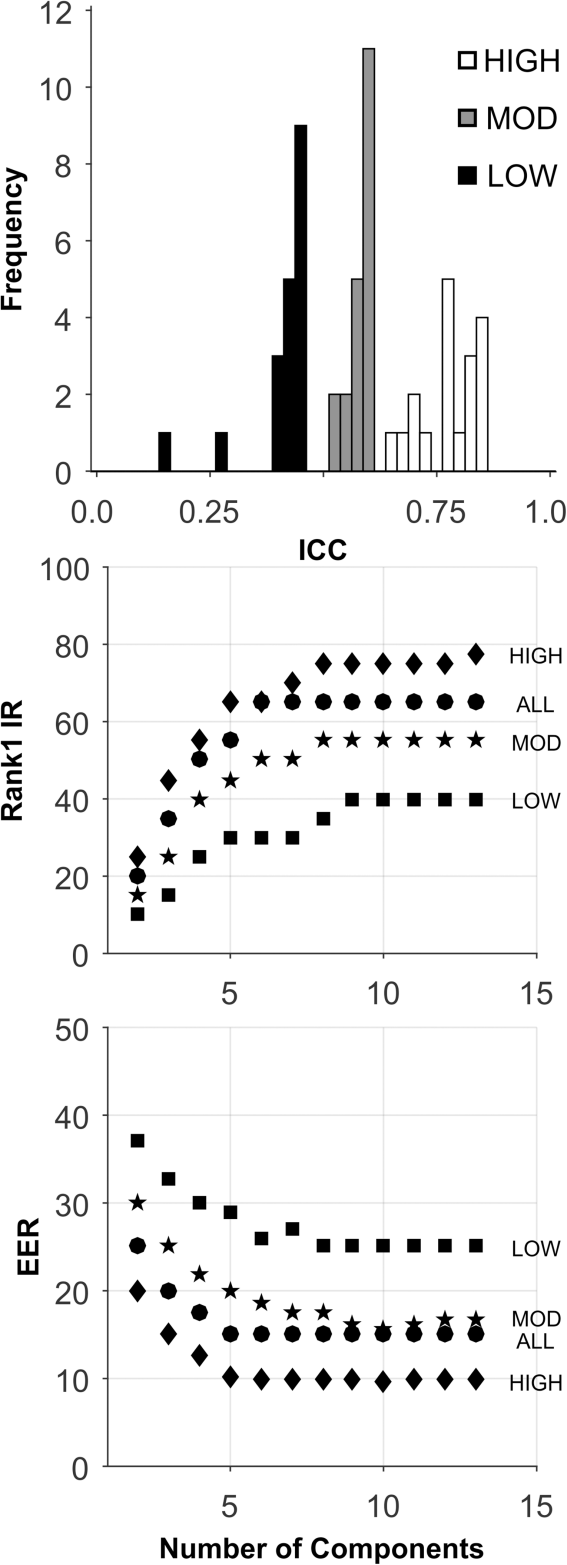
Analysis of the FACE1 database. See caption for Fig 2.

The analyses for this database are presented in Fig 14, and Table 5. There was a wide range of ICCs for these measures (Fig 14, Top). The HIGH ICC set had superior performance both for Rank-1-IR (Fig 14, Middle) and EER (Fig 14, Bottom). Considering the simplicity of the algorithm employed for face recognition, the performance is quite good.

### FACE2 database

**Fig 15:**
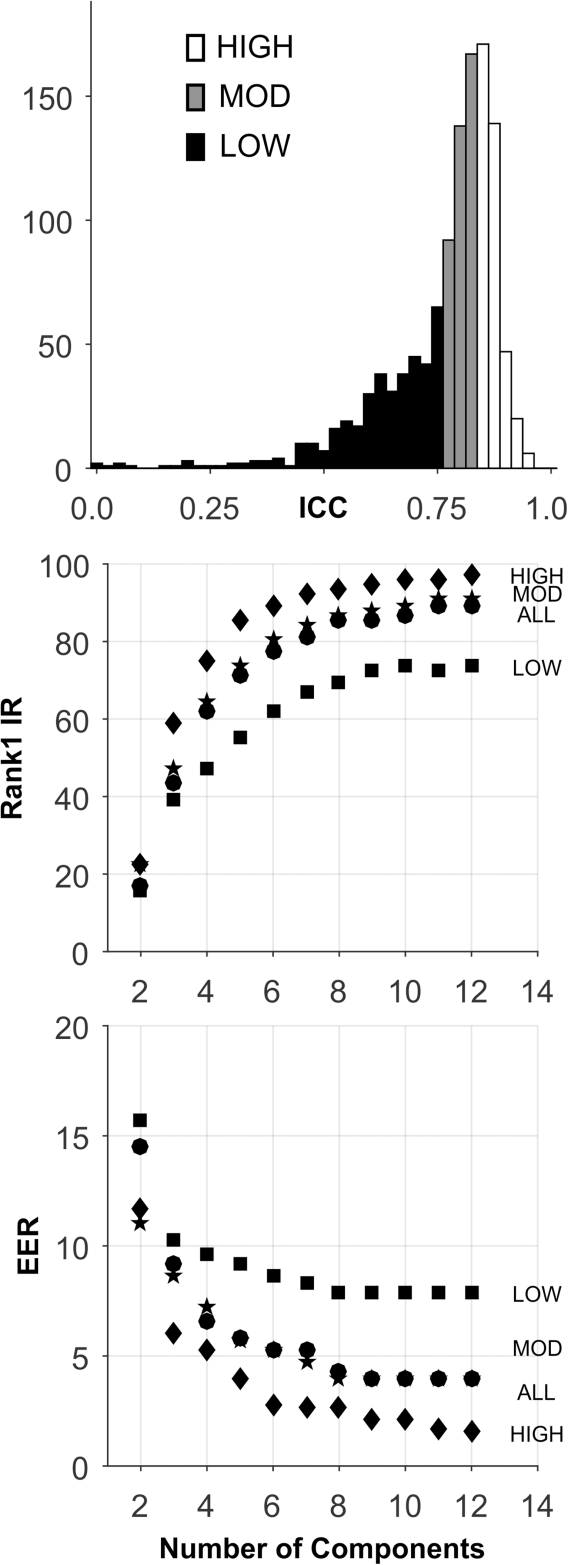
Analysis of the FACE2 database. See caption for Fig 2.

The analyses for this database are presented in Fig 15, and Table 5. The distribution of ICCs was skewed toward higher values, and most of the ICCs were in the 0.5 to 1.0 range (Fig 15, Top). The HIGH ICC set had superior performance both for Rank-1-IR (Fig 14, Middle) and EER (Fig 14, Bottom). Considering how similar the faces in this set were over repeat sessions, it is not surprising that the performance is excellent.

### Brain database

**Fig 16:**
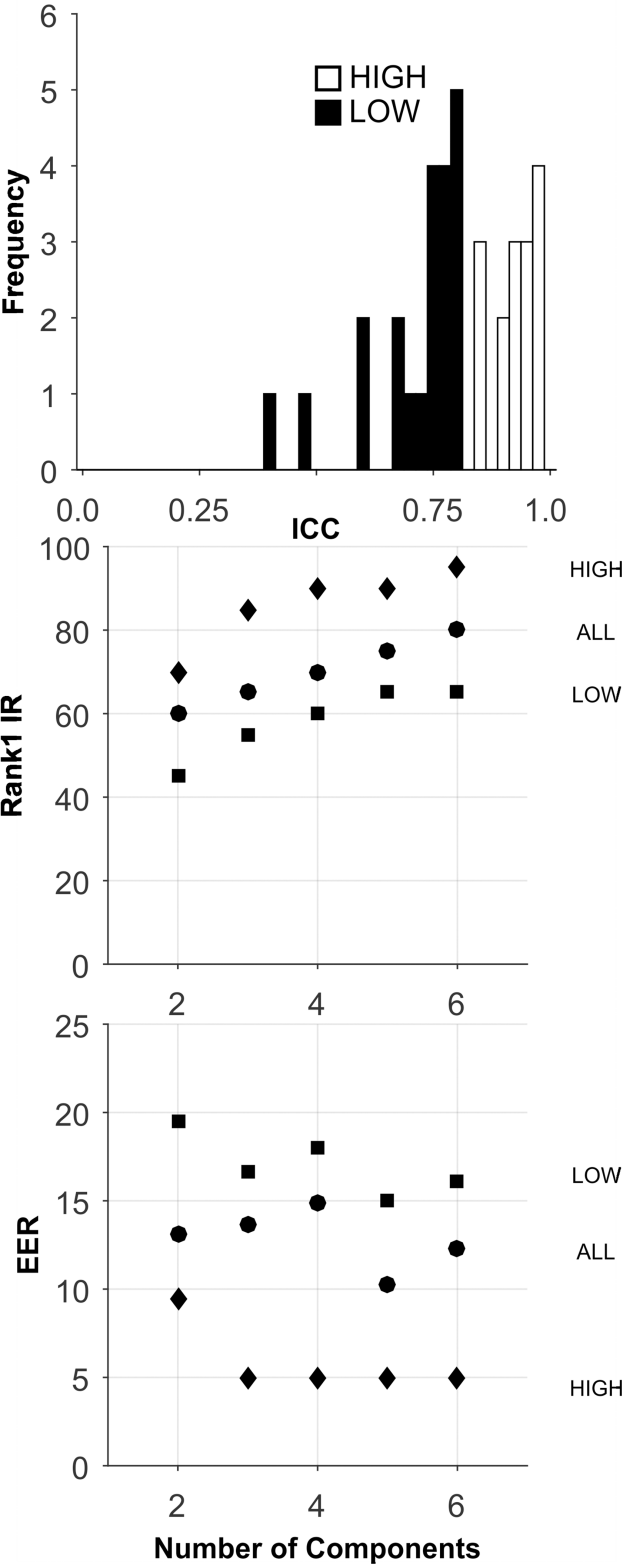
Analysis of the BRAIN database. See caption for Fig 2.

The analyses for this database are presented in Fig 16, and Table 5. There were 37 features for this database, and the ICCs were generally quite high. The HIGH ICC set achieved superior Rank-1-IR peak performance at 6 components (Fig 16, Middle). For EER, the best performance was for the also for the HIGH ICC set, (Fig 16, Bottom, Table 5). Overall, the performance was reasonably good for this database, considering the small sample of subjects.

### Tests of probability of proportions

In 12 of 14 cases, the Rank-1-IR for the HIGH-ICC set was larger than other databases. This amounts to a proportion of 0.86. The probability of getting a proportion this extreme or more extreme, when the null hypothesis is chance (probability = 0.50) is p = 0. 0065 (one-tailed, Exact Binomial Test). For EER, the HIGH-ICC was lower than other ICC sets in 12/14 cases (p = 0. 0065, one-tailed, Exact Binomial Test).

### Tests across databases

#### ICC vs Rank-1-IR or EER

**Fig 17:**
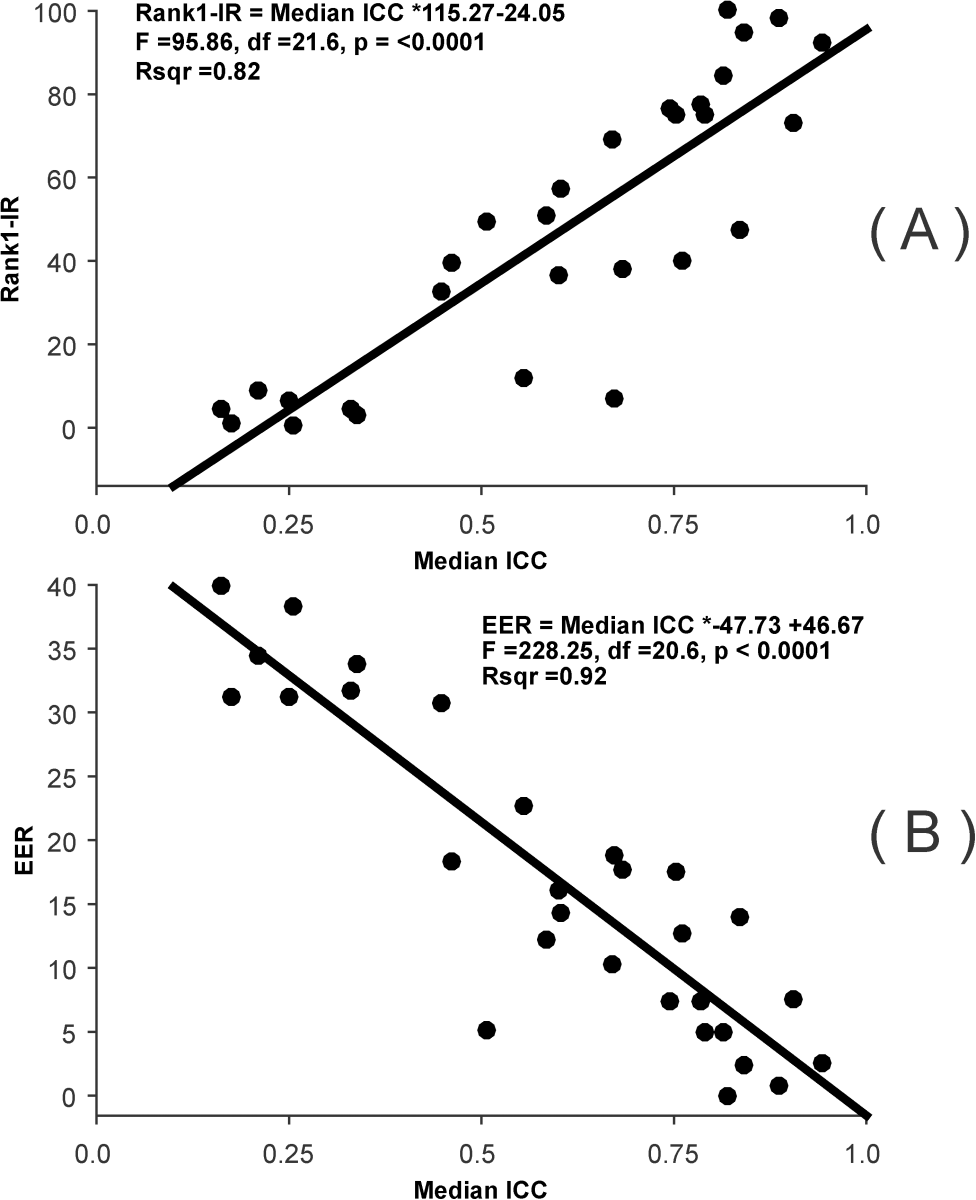
Relationship between biometric performance and ICC across databases. (A) Scatterplot relating Rank-1-IR and median ICC. Note the strong linear relationship. Also shown are the best fitting line, the best estimate of the linear equation, the results of an F-test, and an r^2^ estimate. (B) Scatterplot relating EER and median ICC. This relationship is even stronger than that for Rank-1-IR, with ICC accounting for 92% of the variance in EER.

The results of these analysis are presented in Fig 17A and 17B. There is a very strong linear, statistically significant relationship between both Rank-1-IR (Fig 17A) and EER (Fig 17B), on the one hand, and median ICC, on the other. For Rank-1-IR, we can use the slope and intercept to determine the predicted Rank-1-IR for any ICC. For example, for an increase in ICC from, say, 0.5 to 0.75, we would expect a Rank-1-IR increase from 33.5 to 64.2 (28.8 Rank-1-IR points difference). For EER, (Fig 17B) we would expect a decrease from 22.8 (at ICC = 0.5) to 10.9 (at ICC = 0.75).

#### Median similarity scores

**Fig 18:**
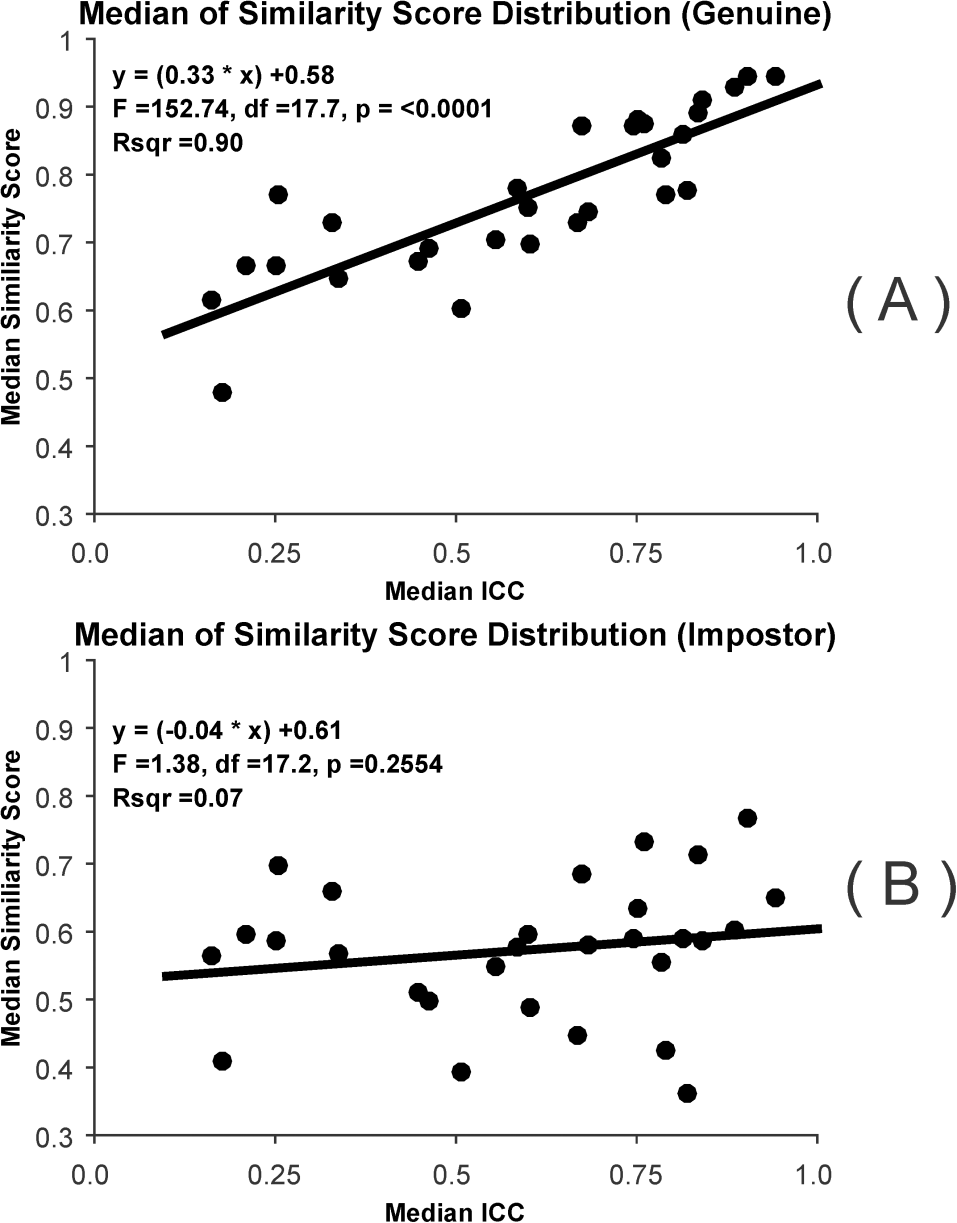
Relationship between the median of the similarity score distribution and ICC. (A) Scatterplot relating the median of the similarity score distribution for the genuine scores and median ICC. Note the strong linear relationship. Also shown are the best fitting line, the best estimate of the linear equation, the results of an F-test, and an r^2^ estimate. (B) Scatterplot relating the median of the similarity score distribution for the impostor scores and median ICC. This relationship is weak and not statistically significant.

The results of these analysis are presented in Fig 18A and 18B. There is a very strong linear, statistically significant relationship between the median similarity score for the genuine distribution and median ICC. The r^2^ for this relationship was 0.90, indicating the ICC accounts for 90% of the variance in median genuine similarity scores. On the other hand, the relationship between the median similarity score for the impostor distribution and median ICC was not statistically significant. The interaction between median ICC and the “Genuine vs Impostor” factor was statistically significant (F = 58.67, df = 1, 42.9, p < 0.001) indicating that the slopes of these relationships were different.

#### IQR similarity scores

**Fig 19:**
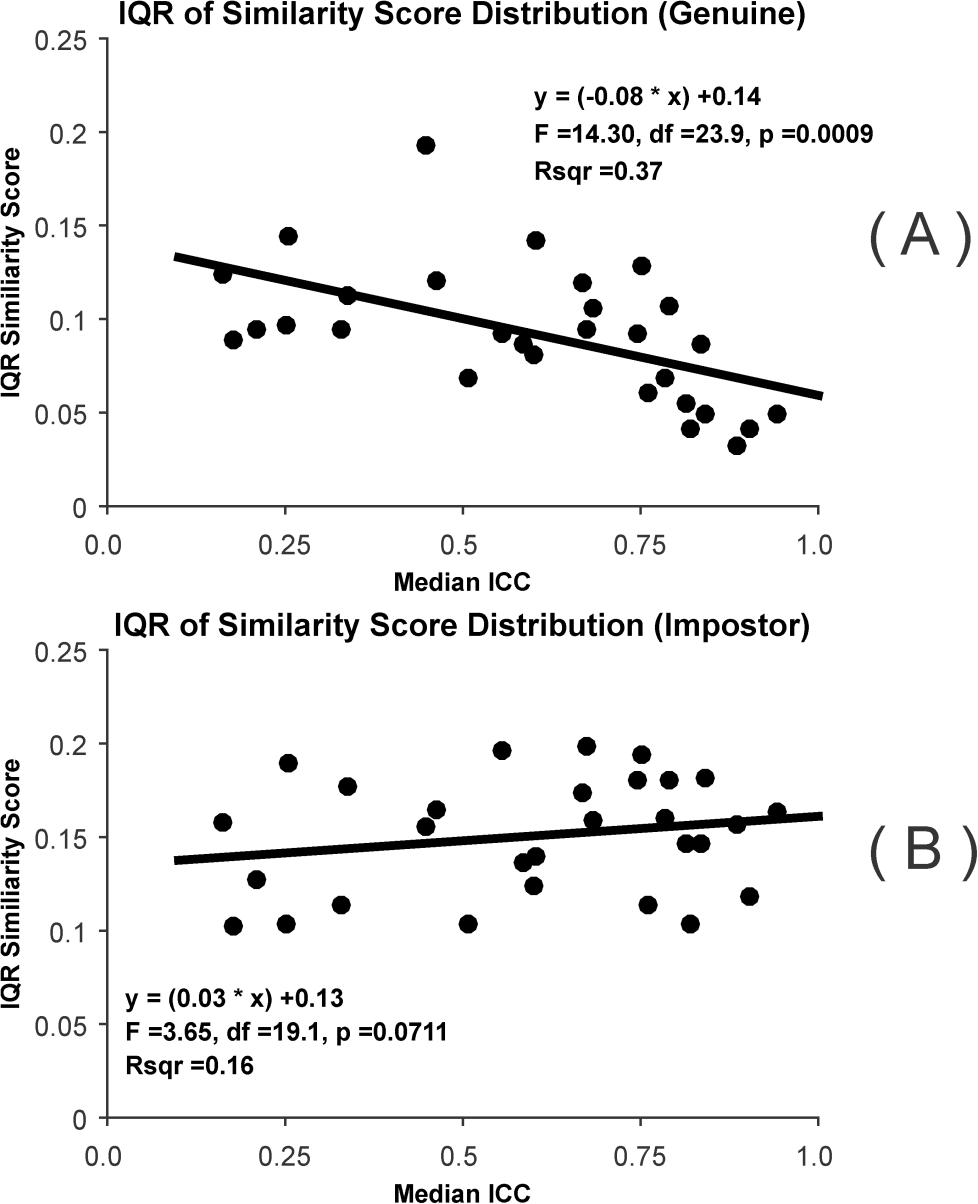
Relationship between the IQR of the similarity score distribution and ICC. (A) Scatterplot relating the IQR of the similarity score distribution for the genuine scores and median ICC. Note the statistically significant linear relationship. Also shown are the best fitting line, the best estimate of the linear equation, the results of an F-test, and an r^2^ estimate. (B) Scatterplot relating the IQR of the similarity score distribution for the impostor scores and median ICC. This relationship is weak and not statistically significant.

The results of these analysis are presented in Fig 19A and 19B. There is a statistically significant relationship between the IQR of the similarity scores for the genuine distribution and median ICC (Fig 19A). However, the r^2^ for this relationship was 0.37, markedly smaller that the r^2^ above, indicating that the ICC is more highly related to the median of the similarity score distribution (genuine scores) than the IQR for the similarity score distribution. The relationship between the IQR of the similarity score for the impostor distribution and median ICC was not statistically significant. The interaction between median ICC and the “Genuine vs Impostor” factor was statistically significant (F = 19.1, df = 1, 43.4, p <0.001) indicating that the slopes of these relationships were different.

## Discussion and conclusion

We have shown that the ICC carries information that is highly relevant to biometrics. We propose to refer to this information as temporal persistence. For Rank-1-IR, in 12 of 14 (p = 00.0065) databases, the HIGH ICC set of features performed better than LOW ICC sets of features, and the MOD (moderate) ICC set of features. For EER, in 12 of 14 (p = 0.0065) databases, the HIGH ICC set of features performed better than LOW ICC sets of features, and the MOD (moderate) ICC set of features. Furthermore, the MOD ICC set of features performed better than the LOW ICC set for the databases where it was created as a separate group (Synthetic, SBA-ST, SBA-LT, FACE1, FACE2). We have documented a strong linear relationship between ICC and either Rank-1-IR or EER across all databases. Furthermore, we have explained why the high ICC groups of features perform so well: Increasing the ICC of a set of measures increases the median of the distribution of genuine similarity scores and, secondarily, decreases the spread (IQR) of these distributions.

The only competition to the excellent performance of the HIGH ICC set was from the ALL ICC groups. What is being compared here is one set, the HIGH ICC set, as a subset of another set, the ALL ICC set. In 8 of 14 databases, this means that the HIGH ICC set employed 50% of the features in the ALL ICC set, and in 5 databases, the HIGH ICC set had only 33% of the features in the ALL ICC set. The implication of this finding is that, not only were HIGH ICC measures better, but that even including lower ICC features with high ICC features leads to decreased biometric performance. So, this not only supports our view on the strength, and importance of the ICC as a measure of temporal persistence for biometric analysis, but supports our specific strategy of dividing the features into different ICC sets, and only using the highest ICC set of features.

This strategy did not work in every case. For the GZVMB database, the ALL set outperformed the HIGH ICC set for both Rank-1-IR and EER. This was a set of highly reliable features. Our strategy depends on a range of ICC values among the features, so it is not surprising that it fails in such a case. There is not much difference between the three sets (LOW, HIGH, ALL) in terms of stability. Since the ALL set has more features, in this case, it performs best.

In one case (CEM-14), the ALL set clearly outperformed the HIGH ICC set for Rank-1-IR. In this case, the database only contained 10 normal features. This result reminds us that a high degree of temporal persistence is not the only important quality for potential biometric features. Obviously, one must have enough independently informative features.

We also have an explanation for why the ICC makes such a difference in most cases. Similarity scores for highly persistent features from the same subjects tested twice (“Genuine Scores”) will have a higher median and a decreased spread (IQR), compared to scores generated from a less stable set. Impostor distributions were not affected much. Either one of these changes (a higher median or a narrower range of similarity scores) for the genuine similarity score distribution will results in increased biometric performance, especially when estimating EER.

We have shown positive results for eye movement, gait, face-recognition and brain-related databases. One may ask if the ICC would be useful for biometric data extracted from fingerprints or irises, or other physical body parts? As noted above, the ICC requires data on an interval or ratio scale that is approximately normally distributed. We simply do not know if features with these characteristics are extracted in such studies or not. If they are, then there is absolutely no doubt that the ICC will index temporal persistence among such features. However, we don’t know how useful such an index would be in such a case. For example, if all such features have ICCs above 0.95, it is probably unlikely that selecting features based on ICC would be useful. But if there are many such features, and there is a wider range of ICCs, and these features are important for discrimination among human subjects, then the strategy employed herein might well yield positive results. Of course, selecting only the more temporally persistent features is not the only strategy. One could, for example, give greater weight in some biometric decision scheme to features with higher reliability. For features which are continuous but not normal one could employ Kendall’s Coefficient of Concordance [12], For features which are dichotomous or polychotomous, either Cohen’s κ or Cohens Weighted κ is will assess the reliability of the ratings over time [15]. Although Cohen’s κ ranges from 0 to 1.0, like the ICC, it is probably not appropriate to directly compare Cohen’s κ values with ICC values.

We would like to invite any reader who might have a database that might be tested with the approach presented herein, to either conduct this analysis themselves or send it to us for analysis. At a minimum, we will return the results to the contributor, and beyond that we can discuss making all such databases and results available on a public website and/or co-publish the findings.

The biometric performance achieved with the HIGH ICC set from the SBA-Short-Term database was the best we have achieved in our laboratory (Table 6). Although we came close to the present results with our Multi-Source Fusion technique [50], it is important to keep in mind that the analysis of Rigas et al., [50] was based on much more data, was much more complicated, and involved fusion at the stimulus level (Poetry reading, and other tasks), and fusion of multiple biometric algorithms. Table 6 also contains the best performance from another laboratory that we are aware of [51]. These researchers were the winners in the “BioEye 2015: Competition on Biometrics via Eye Movements” [52]. Our results and their results are quite similar, but with the special procedures explained above, we could do slightly better. Therefore, we believe that the results we report here are the best performance results ever achieved with eye-movements.

**Table 6:** Published performance for other EM-related approaches.

Authors	Year	Reference	Approach	Best EER Achieved	Best Rank-1 IR Achieved
I. Rigas, O.V. Komogortsev	2014	[53]	Fixation Density Map	10.8	51.0%
O.V. Komogortsev, A. Karpov and C. Holland	2016	[54]	Oculomotor Plant Characteristics	14.0	24.7%
I. Rigas, O.V. Komogortsev and R. Shadmeher	2016	[55]	CEM-B + Acceleration + Vigor	10.6	64.3%
I. Rigas, E. Abdulin and O.V. Komogortsev	2016	[50]	Multi-Source Fusion	5.8	88.6%
A. George and A. Routray	2016	[51]	Gaussian Radial Basis Function & Score Fusion	2.59	89.54
Present results for SBA-ST			HIGH ICC set (122 features, 22 PCA components)	2.7	90.6%
Present results for SBA-ST			With Special Procedures Described in Text	2.0 2.4	91.95 92.62

In the present study, the strategy employed was to divide up the databases into equal sized subsets based on ICC. This is not the only way to employ the ICC for biometrics. Another way would be to try sub-setting the entire set of measures with a range of ICC thresholds (e.g., ICC > 0.3, ICC > 0.4 … ICC > 0.9) and compare biometric performance for these subsets. Our next paper in this line of research will pursue that strategy.

It is somewhat surprising that prior biometric researchers have not employed the ICC to assess temporal persistence, since, in our view, this coefficient is high suitable for biometrics. Furthermore, it was introduced by R.A. Fisher in 1925 [4], and it has been employed in nearly 9,000 published studies according to the National Library of Medicine (PubMed).

The median reliability of the gait-related features was much higher than the eye-movement-related features. This is a small sample of studies for which to reach any firm conclusions on this topic. However, one possibility may be related to the fact that the participants in the gait-related research were practiced before testing: “…the treadmill training and filming took place after each subject had first walked outdoors, and then inside on the laboratory track.” (pg. 67, [39]). It might be worth evaluating the effect of task practice on subsequent test-retest reliability for the eye-movement studies.

In the present case, the reliability of features was assessed in the same sample of subjects used for biometric performance assessment. This is not an ideal situation. In the ideal case, the reliability of features would be established in a distinct sample from that employed in biometric assessment. Also, although the split-sample approach is a highly efficient research tool, testing the biometric performance in completely novel subject samples will eventually be required. Nonetheless, our results illustrate that temporal persistence (stability) is critically important for biometric feature selection, and performance, and that the ICC is an excellent tool to assess it.

## Supporting information

S1 Document The calculation of the intraclass correlation coefficient. (DOCX)

S2 Document Basic statistics and accuracy estimates. (DOCX)

## References

[1] Littell RC. SAS for mixed models 2nd ed. Cary, N.C.: SAS Institute, Inc.; 2006. xii, 814 p. p.

[2] Shrout PE, Fleiss JL. Intraclass correlations: uses in assessing rater reliability. Psychol Bull. 1979;86(2):420–8. 18839484 10.1037//0033-2909.86.2.420

[3] McGraw KO, Wong SP. Forming inferences about some intraclass correlation coefficients. Psychological methods. 1996;1(1):30.

[4] Fisher RA. Statistical methods for research workers Edinburgh, London,: Oliver and Boyd; 1925.

[5] Mahbubur Rahman SM, Lata SP, Howlader T. Bayesian face recognition using 2D Gaussian-Hermite moments. EURASIP Journal on Image and Video Processing. 2015;2015(1):1–20.

[6] Cicchetti DV. The precision of reliability and validity estimates re-visited: distinguishing between clinical and statistical significance of sample size requirements. J Clin Exp Neuropsychol. 2001;23(5):695–700. doi: 10.1076/jcen.23.5.695.1249 11778646 10.1076/jcen.23.5.695.1249

[7] Cicchetti DV, Sparrow SA. Developing criteria for establishing interrater reliability of specific items: applications to assessment of adaptive behavior. Am J Ment Defic. 1981;86(2):127–37. 7315877

[8] Selkainaho K, Pakkinen J, editors. ICC statistic as criterion for classification and feature selection. Pattern Recognition, 1988, 9th International Conference on; 1988 14–17 Nov 1988.

[9] Cohn JF, Schmidt K, Gross R, Ekman P, editors. Individual differences in facial expression: Stability over time, relation to self-reported emotion, and ability to inform person identification. Proceedings of the 4th IEEE International Conference on Multimodal Interfaces; 2002: IEEE Computer Society.

[10] Napflin M, Wildi M, Sarnthein J. Test-retest reliability of resting EEG spectra validates a statistical signature of persons. Clin Neurophysiol. 2007;118(11):2519–24. doi: 10.1016/j.clinph.2007.07.022 17892969 10.1016/j.clinph.2007.07.022

[11] Tome P, Vera-Rodriguez R, Fierrez J, Ortega-Garcia J. Facial soft biometric features for forensic face recognition. Forensic Sci Int. 2015;257:271–84. doi: 10.1016/j.forsciint.2015.09.002 26454196 10.1016/j.forsciint.2015.09.002

[12] Kendall MG, Smith BB. The Problem of m Rankings. 1939:275–87.

[13] Ulery BT, Hicklin RA, Buscaglia J, Roberts MA. Repeatability and reproducibility of decisions by latent fingerprint examiners. PLoS One. 2012;7(3):e32800 doi: 10.1371/journal.pone.0032800 22427888 10.1371/journal.pone.0032800 PMC3299696

[14] Bartko JJ. The intraclass correlation coefficient as a measure of reliability. Psychol Rep. 1966;19(1):3–11. doi: 10.2466/pr0.1966.19.1.3 5942109 10.2466/pr0.1966.19.1.3

[15] Bartko JJ, Carpenter WT Jr. On the methods and theory of reliability. J Nerv Ment Dis. 1976;163(5):307–17. 978187 10.1097/00005053-197611000-00003

[16] Dror I, Rosenthal R. Meta‐analytically quantifying the reliability and biasability of forensic experts. Journal of Forensic Sciences. 2008;53(4):900–3. doi: 10.1111/j.1556-4029.2008.00762.x 18489557 10.1111/j.1556-4029.2008.00762.x

[17] Lanitis A. A survey of the effects of aging on biometric identity verification. International Journal of Biometrics. 2010;2(1):34.

[18] Scheidat T, Heinze J, Vielhauer C, Dittmann J, Kraetzer C. Comparative review of studies on aging effects in context of biometric authentication SPIE-IS&T 2011;7881.

[19] Czajka A. Influence of iris template aging on recognition reliability. Communications in Computer and Information Science 2014.

[20] Modi SK, Elliott SJ, Kim H, editors. Statistical analysis of fingerprint sensor interoperability performance. Proceeding of the IEEE 3rd International Conference on Biometrics: Theory, Applications, and Systems (BTAS); 2009 28–30 Sept. 2009.

[21] Ramana N, Chellappa R. Face Verification Across Age Progression. IEEE Transactions on Image Processing. 2006;15(11):3349–61. 17076395 10.1109/tip.2006.881993

[22] Ling H, Soatto S, Ramanathan N, Jacobs DW, editors. A Study of Face Recognition as People Age. 2007 IEEE 11th International Conference on Computer Vision; 2007 14–21 Oct. 2007.

[23] Shimon K. Modi DSJE. Impact of Image Quality on Performance: Comparison of Young and Elderly Fingerprints. Proceedings of the 6th International Conference on Recent Advances in Soft Computing (RASC 2006). 2006:449–54.

[24] J. W. Carls RR, M. Grimaila, S. Rogers. Biometric enhancements: Template aging error score analysis. 8th IEEE International Conference on Automatic Face & Gesture Recognition. 2008:1–8.

[25] Geng X, Zhou ZH, Smith-Miles K. Automatic Age Estimation Based on Facial Aging Patterns. IEEE Transactions on Pattern Analysis and Machine Intelligence. 2007;29(12):2234–40. 10.1109/TPAMI.2007.70733 17934231

[26] Lanitis A. Comparative Evaluation of Automatic Age Progression Methodologies. EURASIP Journal on Advances in Signal Processing. 2008;2008(1):239480.

[27] Lanitis A, Tsapatsoulis N. Quantitative evaluation of the effects of aging on biometric templates. IET Computer Vision. 2011;5(6):338.

[28] Park U, Tong Y, Jain AK. Age-invariant face recognition. IEEE Trans Pattern Anal Mach Intell. 2010;32(5):947–54. doi: 10.1109/TPAMI.2010.14 20299717 10.1109/TPAMI.2010.14

[29] Tobias Scheidat KK, Vielhauer Claus. Short Term Template Aging Effects on Biometric Dynamic Handwriting Authentication Performance. IFIP International Federation for Information Processing 2012 2012:107–16.

[30] Javier Galbally MM-D, Fierrez Julian. Aging in Biometrics: An Experimental Analysis on On-Line Signature. PLoS ONE. 2013;8(7):e69897 doi: 10.1371/journal.pone.0069897 23894557 10.1371/journal.pone.0069897 PMC3720939

[31] Ortiz E, Bowyer KW, Flynn PJ, editors. A linear regression analysis of the effects of age related pupil dilation change in iris biometrics. 2013 IEEE Sixth International Conference on Biometrics: Theory, Applications and Systems (BTAS); 2013 Sept. 29 2013-Oct. 2 2013.

[32] Trokielewicz M, editor Linear regression analysis of template aging in iris biometrics. 3rd International Workshop on Biometrics and Forensics (IWBF 2015); 2015 3–4 March 2015.

[33] Fenker SP, Ortiz E, Bowyer KW. Template Aging Phenomenon in Iris Recognition. IEEE Access. 2013;1:266–74.

[34] Manjani I, Sumerkan H, Flynn PJ, Bowyer KW, editors. Template aging in 3D and 2D face recognition. 2016 IEEE 8th International Conference on Biometrics Theory, Applications and Systems (BTAS); 2016 6–9 Sept. 2016.

[35] Rigas IF, L., and Komogortsev, O.,. A Study on the Extraction and Analysis of a Large Set of Eye Movement Features during Reading. ArXiv e-prints. 2017;1703.09167.

[36] Holland CD, Komogortsev OV, editors. Complex eye movement pattern biometrics: Analyzing fixations and saccades. 2013 International Conference on Biometrics (ICB); 2013.

[37] Komogortsev O, Holland C, Karpov A, Price LR. Biometrics via Oculomotor Plant Characteristics: Impact of Parameters in Oculomotor Plant Model. ACM Trans Appl Percept. 2014;11(4):1–17.

[38] Holland CD, Komogortsev OV. Complex Eye Movement Pattern Biometrics: The Effects of Environment and Stimulus. IEEE Transactions on Information Forensics and Security. 2013;8(12):2115–26.

[39] Shutler JD, Grant MG, Nixon MS, Carter JN. On a large sequence-based human gait database Applications and Science in Soft Computing: Springer Berlin Heidelberg; 2004 p. 339–46.

[40] Jeff P. Foster MSN, Pr€ugel-Bennett Adam. Automatic gait recognition using area-based metrics. Pattern Recognition Letters. 2003;24:2489–97.

[41] Shutler JD, Nixon MS, editors. Zernike Velocity Moments for Description and Recognition of Moving Shapes. BMVC; 2001.

[42] Iscan Z, Jin TB, Kendrick A, Szeglin B, Lu H, Trivedi M, et al Test-retest reliability of freesurfer measurements within and between sites: Effects of visual approval process. Hum Brain Mapp. 2015;36(9):3472–85. PubMed Central PMCID: PMCPMC4545736. doi: 10.1002/hbm.22856 26033168 10.1002/hbm.22856 PMC4545736

[43] SR-Research. EyeLink 1000 http://www.sr-research.com/EL_1000.html2015 [07/2015].

[44] Nyström M, Holmqvist K. An adaptive algorithm for fixation, saccade, and glissade detection in eyetracking data. Behavior Research Methods. 2010;42(1):188–204. doi: 10.3758/BRM.42.1.188 20160299 10.3758/BRM.42.1.188

[45] Prokop RJ, Reeves AP. A survey of moment-based techniques for unoccluded object representation and recognition. CVGIP: Graph Models Image Process. 1992;54(5):438–60.

[46] Fischl B, Salat DH, Busa E, Albert M, Dieterich M, Haselgrove C, et al Whole brain segmentation: automated labeling of neuroanatomical structures in the human brain. Neuron. 2002;33(3):341–55. 11832223 10.1016/s0896-6273(02)00569-x

[47] Winer BJ, Brown DR, Michaels KM. Statistical Principles in Experimental Design: McGraw-Hill; 1991.

[48] Tabachnick BG, Fidell LS. Using multivariate statistics 6th ed. Boston: Pearson Education; 2013. xxxi, 983 p. p.

[49] Johnson DE. Applied multivariate methods for data analysts Pacific Grove, Calif.: Duxbury Press; 1998. xiv, 567 p. p.

[50] Rigas I, Abdulin E, Komogortsev O. Towards a multi-source fusion approach for eye movement-driven recognition. Information Fusion. 2015.

[51] George A, Routray A. A score level fusion method for eye movement biometrics. Pattern Recognition Letters. 2016;82:207–15.

[52] Komogortsev OV, Rigas I, editors. BioEye 2015: Competition on biometrics via eye movements. 2015 IEEE 7th International Conference on Biometrics Theory, Applications and Systems (BTAS); 2015 8–11 Sept. 2015.

[53] Ioannis Rigas OVK. Biometric recognition via fixation density maps. 2014;Conference Paper in Proceedings of SPIE—The International Society for Optical Engineering 9075.

[54] Komogortsev OV, Karpov A, Price L, Aragon C, editors. Biometric Authentication via Oculomotor Plant Characteristic. IEEE/IARP International Conference on Biometrics (ICB); 2012.

[55] Rigas I, Komogortsev O, Shadmehr R. Biometric recognition via eye movements: Saccadic vigor and acceleration cues. ACM Transactions on Applied Perception (TAP). 2016;13(2):6.

