# Supplementary material for: Method to assess the temporal persistence of potential biometric features: Application to oculomotor, gait, face and brain structure databases
Source: PLoS One. 2017 Jun 2;12(6):e0178501. doi: 10.1371/journal.pone.0178501 (PMC5456116; doi:10.1371/journal.pone.0178501)
Supplement: S1 Document — (DOCX) [file pone.0178501.s001.docx]

S1_Document – The Calculation of the ICC

Two approaches

The concept of the ICC is firmly rooted in the concepts of the analysis of variance (ANOVA), including Mixed Models and variance components maximum likelihood-based (VCML) analysis. It would be difficult to understand what is happening without some experience with these statistical methods. A good introductory statistical text [1-3] will help here. Variance components analysis is covered in [3, 4].

There are two major methods to calculate the ICC: (1) ANOVA-based, and (2) VCML-based. The ANOVA-based approach is the classic approach and it is clearly presented in two key references: Shrout and Fliess (SF) [5] and McGraw and Wong [6]. Let’s start off with an example from Shrout and Fleiss [5] (Table 2 in their paper), (SF Data), assuming 6 subjects (“Targets” in SF nomenclature) and 4 sessions (“Judges” in SF nomenclature). There are two ways to arrange this data, the wide form:

| S1_Document_Table 1: Shrout and Fleiss Example in  Wide Data Format | | | |
| --- | --- | --- | --- |
| Session1 | Session2 | Session3 | Session4 |
| 9 | 2 | 5 | 8 |
| 6 | 1 | 3 | 2 |
| 8 | 4 | 6 | 8 |
| 7 | 1 | 2 | 6 |
| 10 | 5 | 6 | 9 |
| 6 | 2 | 4 | 7 |

And Long Form:

| S1_Document_Table 2: Long Form | | |
| --- | --- | --- |
| Subject | Session | Measure |
| 1 | 1 | 9 |
| 2 | 1 | 6 |
| 3 | 1 | 8 |
| 4 | 1 | 7 |
| 5 | 1 | 10 |
| 6 | 1 | 6 |
| 1 | 2 | 2 |
| 2 | 2 | 1 |
| 3 | 2 | 4 |
| … | … | … |
| 5 | 4 | 9 |
| 6 | 4 | 7 |

We now need to introduce the concept of fixed effects and random effects. Let’s assume that we start with data in the long form. In the context of this data structure, the issue is whether you are interested in the reliability of these specific subjects versus a general population of subjects from which you draw your sample. In the first case, you would model the subject effect as a fixed effect and in the second case you would model the subject effect as a random effect. Almost always, investigators are interested in generalizing from the subjects in the study to the population from which they are drawn, and therefore the subject effect is considered random. Similar considerations also apply to the session effect. If you are interested in the reliability of these specific sessions, then you would model the session effect as a fixed effect. A distinct ICC calculation for this case is provided for as “case 3” in Shrout and Fleiss [5]. But if you are interested in generalizing to a population of sessions from which you have drawn your sessions, then you would model the session effect as a random effect. In our view, the most common situation is that the investigators are interested in making conclusions about populations of sessions (raters, judges), and so both the session effect and the subject effect are modelled as a random effect. So, for our ANOVA, we have a two-way Random Effects model. In this model, the intercept (Grand Mean) is still modelled as a fixed effect.

When the Shrout and Fleiss [5] paper was written, software had not developed to the point where random effects could be estimated directly using a VCML-based method, so one typically performed a fixed effect ANOVA (using data in the long form), and used the mean squares of that analysis to compute variance estimates for the random subject effect and the random session effect. Here is a fixed effects ANOVA table for the analysis the SF data:

| S1_Document_Table 3:  Fixed Effect ANOVA for SF Data | | | | |
| --- | --- | --- | --- | --- |
| Source | DF | Sum of Squares | F Ratio | Prob > F |
| Subject | 5 | 56.21 | 11.03 | 0.0001 |
| Session | 3 | 97.46 | 31.87 | 0.0001 |
| Error | 15 | 15.29 | 1.02 |  |

To use this fixed effects ANOVA to compute variance due to Subjects and variance due to Sessions as random effects, and variance due to Error, we need to calculate the Mean Squares (MS) (Sum of Squares divided by degrees of freedom) for these effects:

MS-Subjects = 56.21 / 5 = 11.24

MS-Sessions = 97.46 / 3 = 32.49

MS-Error = 15.29 / 15 = 1.02

We also need to define k as the number of sessions (in this case 4) and the n as number of subjects (in this case 6):

k=4, n=6

The variance due to subjects, modelling subjects as a random effect, is:

(MS-Subjects-MS-Error)/k = (11.24 –1.02)/4 = 2.56

The variance due to sessions, modelling sessions as a random effect, is:

(MS-Sessions-MS-Error)/n = (32.49 – 1.02)/6 = 5.24

The variance due to error is MS_Error = 1.02

Total Variance is the sum of variance due to subjects + variance due to sessions + variance due to error. This comes to 2.56+5.24+1.02=8.82,

The ICC is simply the variance due to subjects (2.56) divided by total variance (8.82) = 0.29.

Different equations are required for the case where the investigators are interested in only the sessions (or raters) studied, and the session effect is modelled as a fixed effect.

One can use the ICC function for MATLAB [7] ([Matlab ICC function](https://www.mathworks.com/matlabcentral/fileexchange/22099-intraclass-correlation-coefficient--icc-)) to calculate the ICC. It expects data in the wide format, without column headers. To get the ICC:

**% Read in the Data**

>a = csvread('ShroutAndFliessDataSample_WideForm.csv',1,0)

**% Run the function**

>ICC(a,'A-1')

ans = 0.2898

Using the r language [8], one cat get the ICC using the icc function in the irr package [9]. This also expects data in the wide format:

**# load the library**

library(“irr”)

**# read in the data**

mydata <- read.csv('ShroutAndFliessDataSample_WideForm.csv')

**# run the function**

myICC < -irr::icc(mydata, model="twoway",type="agreement", unit="single")

**# print the result**

print(myICC)

>ICC(A,1) = 0.29

In SPSS, go to “Analysis>Scale>Reliability”, move Session1 to Session4 to the “items” box, select statistics and check mark “Intraclass correlation coefficient”, choose a “Two-Way Random” model and Absolute Agreement as the type. The ICC you want is in the “Single Measures” row. There are a number of other statistical packages that can compute the ICC using this ANOVA fixed effects format.

However, if one is interested in a two-way, random effects model, as most are, then there is a more direct method for calculating the ICC using a VCML analysis. With this approach, there is no need to perform an ANOVA. VCML can estimate the variance due to subjects, the variance due to session, and the residual variance directly. If we define total variance as the sum of variance due to subjects + variance due to sessions + residual variance, then the ICC we want is simply variance due to subjects divided by total variance.

For VCML analysis, the variances estimates are the result of an estimation procedure. The algorithm uses the Newton-Raphson algorithm, and iterates until the log-likelihood objective function converges [4]. When the data are balanced (equal N samples per session) the ANOVA method and the variance-components generally produce identical results. However, there are cases in which the ANOVA-based method will produce variance estimates that are less than 0 (an impossible result). The variance components analysis will never produce a negative variance estimate. If there is missing data, the standard ANOVA methods simply remove all data from that subject (listwise deletion). There are special procedures for estimating all the necessary variances from a two-way random effects model using ANOVA with missing data (See Chapter 5, [4]), but this is seldom done, since the VCML approach effortlessly estimates all variance components, using all available data. Thus the variance components procedure is more robust than the ANOVA procedure in the face of missing data.

We are not aware of a VCML procedure in MATLAB, but a variance components analysis can be performed using the r procedure “varComp” in in the varComp r package [10]. SPSS [11] and SAS [12] have variance components programs, as does JMP [13].

In the present paper, we use the tools in r. The data are in the long form. Here are some r commands:

**# Read the data:**

mydata <- read.csv('ShroutAndFliessDataSample_LongForm.csv')

**# Inform r that subjects and sessions are to be treated as factors:**

mydata$Subject <- as.factor(mydata$Subject)

mydata$Session <- as.factor(mydata$Session)

**# Load the Variance Components Package**

Library(varComp)

**# Run the Variance Components Analysis**

a<-varComp(Measure ~ 1, mydata, ~ Subject + Session)

**# Extract the variance estimates from the results**

VarSubject <- as.numeric(a$varComps[1])

VarSession <- as.numeric(a$varComps[2])

VarError <- a$sigma2

TotalVariance = VarSubject + VarSession + VarError

ICC = VarSubject/TotalVariance

cat(sprintf("ICC from Variance Components Analysis= %0.2f\n", ICC))

We prefer the variance components method for its advantages (it will not produce a negative variance estimate, it is more robust to missing data), and since it is more elegant, and direct. In Fig. 1 of the main manuscript, we provide variance components estimates below the Pearson r and ICC calculations. These were created using the variance components approach.

References

1. Hays WL. Statistics. 5th ed. Fort Worth: Harcourt College Publishers; 1994. xviii, 1112 p. p.

2. McCulloch CE, Searle SR, Neuhaus JM. Generalized, linear, and mixed models. 2nd ed. Hoboken, N.J.: Wiley; 2008. xxv, 384 p. p.

3. Milliken GA, Johnson DE. Analysis of messy data. 2nd ed. Boca Raton: CRC Press; 2009. v. <1- > p.

4. Searle SR, Casella G, McCulloch CE. Variance components. New York: Wiley; 1992. xxiii, 501 p. p.

5. Shrout PE, Fleiss JL. Intraclass correlations: uses in assessing rater reliability. Psychol Bull. 1979;86(2):420-8. PubMed PMID: 18839484.

6. McGraw KO, Wong SP. Forming inferences about some intraclass correlation coefficients. Psychological methods. 1996;1(1):30.

7. The MathWorks I. MATLAB 8.5 (2015a). Natick, MA, USA: The MathWorks, Inc.; 2015.

8. Team RC. R: A Language and Environment for Statistical Computing. Vienna, Austria: R Foundation for Statistical Computing; 2016.

9. Matthias Gamer JL, Ian Fellows, Puspendra Singh. irr: Various Coefficients of Interrater Reliability and Agreement (R package version 0.84)2012.

10. Qu L. varComp: Variance Component Models. R package version 01-360. 2015.

11. IBM Corp. IBM SPSS Statistics for Windows, Version 22.0. Armonk, NY: IBM Corp.; 2013.

12. Institute S. SAS 9.3 (SAS Institute, Cary NC).". Cary, NC: SAS Insitute; 2011.

13. SAS Institute I. JMP PRO 12 <x>. SAS Institute Inc., Cary, NC, 1989-2007. Cary, NC: SAS Institute, Inc.; 2016.
