# Supplementary material for: Method to assess the temporal persistence of potential biometric features: Application to oculomotor, gait, face and brain structure databases
Source: PLoS One. 2017 Jun 2;12(6):e0178501. doi: 10.1371/journal.pone.0178501 (PMC5456116; doi:10.1371/journal.pone.0178501)
Supplement: S2 Document — (DOCX) [file pone.0178501.s002.docx]

**S2_Document – Basic Statistics and Accuracy Estimates**

The short-term eye-movement datasets were all from what we call “Round 1”, which is the first time we recorded these subjects. It consists of 2 sessions, approximately 19 minutes apart. The long-term eye-movement datasets contrasted Round 1 Session 1 with Round 5 Session 1. Round 5 was approximately 11 months later.

As noted in the main text, we only analyzed the portion of the poetry reading task during which subjects were actually reading. In the first row of the Table, we see the statistics on time spend reading for Round 1 Session 1, Round 1 Session 2, and Round 5 Session 1.

The Eyelink 1000 automatically detects blinks and replaces the signal values during blinks with NaNs (Not a Number). In row 2 of the Table below, we present the percent of recording that is NaN. In the third row, we present the number of blinks in each recording. We also computed the rate of blinks per second. Finally, the positional accuracy of each recording for each subject is provided by the Eyelink 1000 device. Spatial Accuracy statistics are presented in row 5 of the Table.

|  | Supplementary Document 2 - Table | | | | | | | | | | | |
| --- | --- | --- | --- | --- | --- | --- | --- | --- | --- | --- | --- | --- |
|  | Round 1 Session 1 (N=298) | | | | Round 1 Session 2 (N=298) | | | | Round 5 Session 1 (N=68) | | | |
|  | Mean | SD | Min | Mean | Mean | SD | Min | Max | Mean | SD | Min | Max |
| Time Spent Reading (sec) | 49.62 | 9.31 | 20.00 | 60.00 | 50.85 | 8.41 | 22.42 | 60.00 | 49.91 | 9.14 | 21.42 | 60.00 |
| Percent of Recording that is NaN | 2.50 | 2.73 | 0.00 | 14.36 | 3.33 | 3.98 | 0.00 | 27.72 | 2.98 | 4.64 | 0.00 | 34.56 |
| Number Of Blinks | 10.56 | 7.63 | 1.00 | 45.00 | 12.24 | 8.90 | 1.00 | 45.00 | 12.42 | 8.64 | 1.99 | 39.84 |
| Blinks Per Second | 0.21 | 0.82 | 0.02 | 0.813 | 0.24 | 1.06 | 0.02 | 0.905 | 0.25 | 0.17 | 0.03 | 0.73 |
| Spatial Accuracy | 0.50 | 0.17 | 0.20 | 1.06 | 0.49 | 0.17 | 0.20 | 1.19 |  |  |  |  |
